# The Rapid Methylation of T-DNAs Upon *Agrobacterium* Inoculation in Plant Leaves

**DOI:** 10.3389/fpls.2019.00312

**Published:** 2019-03-15

**Authors:** Joshua G. Philips, Kevin J. Dudley, Peter M. Waterhouse, Roger P. Hellens

**Affiliations:** ^1^Centre for Tropical Crops and Biocommodities, Queensland University of Technology, Brisbane, QLD, Australia; ^2^Institute for Future Environments, Central Analytical Research Facility, Queensland University of Technology, Brisbane, QLD, Australia; ^3^Institute for Future Environments, Queensland University of Technology, Brisbane, QLD, Australia; ^4^Department of Biochemistry, University of Otago, Dunedin, New Zealand

**Keywords:** *de novo* methylation, epigenetics, *Nicotiana benthamiana*, PTGS, T-DNA, TGS, transient, intron-mediated enhancement

## Abstract

*Agrobacterium tumefaciens* has been foundational in the development of transgenic plants for both agricultural biotechnology and plant molecular research. However, the transformation efficiency and level of transgene expression obtained for any given construct can be highly variable. These inefficiencies often require screening of many lines to find one with consistent and heritable transgene expression. Transcriptional gene silencing is known to affect transgene expression, and is associated with DNA methylation, especially of cytosines in symmetric CG and CHG contexts. While the specificity, heritability and silencing-associated effects of DNA methylation of transgene sequences have been analyzed in many stably transformed plants, the methylation status of transgene sequences in the T-DNA during the transformation process has not been well-studied. Here we used agro-infiltration of the eGFP reporter gene in *Nicotiana benthamiana* leaves driven by either an AtEF1α-A4 or a CaMV-35S promoter to study early T-DNA methylation patterns of these promoter sequences. The T-DNA was examined by amplicon sequencing following sodium bisulfite treatment using three different sequencing platforms: Sanger sequencing, Ion Torrent PGM, and the Illumina MiSeq. Rapid DNA methylation was detectable in each promoter region just 2–3 days post-infiltration and the levels continued to rapidly accumulate over the first week, then steadily up to 21 days later. Cytosines in an asymmetric context (CHH) were the most heavily and rapidly methylated. This suggests that early T-DNA methylation may be important in determining the epigenetic and transcriptional fate of integrated transgenes. The Illumina MiSeq platform was the most sensitive and robust way of detecting and following the methylation profiles of the T-DNA promoters. The utility of the methods was then used to show a subtle but significant difference in promoter methylation during intron-mediated enhancement. In addition, the method was able to detect an increase in promoter methylation when the eGFP reporter gene was targeted by siRNAs generated by co-infiltration of a hairpin RNAi construct.

## Introduction

Transformation efficiency using *Agrobacterium* can be dependent on many variables including the *Agrobacterium* strain, the host plant species, and the age and tissue type being used. Additional factors influencing the efficiency of T-DNA transfer include, but are not limited to, the optical density of the *Agrobacterium* solution, the concentration of the *vir* gene inducer, acetosyringone, time in co-cultivation media, infiltration/inoculation conditions including addition of surfactants and tissue culture conditions (Clough and Bent, 1998; Jin et al., 2005; Shrawat and Lörz, 2006; Lizamore and Winefield, 2014). The optimization efforts in many different crop species such as the cereals; rice, maize, wheat, barley, sugarcane, sorghum and rye, reviewed by Yang et al. (2000), citrus hybrids Cervera et al. (1998), apple James et al. (1993), and tomato Sharma et al. (2009) demonstrate the challenges faced for transformation efficiency. Once transformed, transgenic expression itself can often be variable through influences such as the T-DNA copy number, T-DNA integration site within the genome, the regulatory sequences used to drive transgene expression and transgene silencing (Butaye et al., 2005).

Variation in transgene expression based on its integration site within the plant genome is often referred to as the ‘position effect.’ It had previously been suggested that T-DNA integration favors transcriptionally active euchromatin regions over transcriptionally inactive heterochromatin (reviewed in Kim and Gelvin, 2007). However, other studies suggest transgene integration may well be random in higher eukaryotes, resulting in integration within both euchromatin and heterochromatin without bias (Forsbach et al., 2003; Kim and Gelvin, 2007; Singer S.D et al., 2012). In addition to the positional effect’s theory, transgene expression can be influenced when integrated near endogenous regulatory elements. These elements can include enhancers or repressors which can influence transgene expression resulting in variable expression patterns. To minimize the effects of variation in expression, matrix-associated regions (MARs) have been included in transgenic constructs. These function by insulating the transgene from condensing chromatin and maintain transcription regardless of the integration site (Singer S.D et al., 2012). When transgenes flanked by a MAR were transformed in tobacco, poplar and maize, variation in transgene expression and spatial patterns between individual transformants was still evident, overall the MAR provided limited success in improving transformation efficiency (van Leeuwen et al., 2000, 2001; Brouwer et al., 2002; Li et al., 2008).

As well as these documented variables that affect transgene expression and transformation efficiency, it is also possible that methylation of the T-DNA during the early stages of plant transformation may play a role. Certainly, in transformed plants, T-DNA methylation has long been shown to be present and to impair T-DNA expression (Gelvin et al., 1983; Amasino et al., 1984). The early methylation of selectable marker and transgene-of-interest promoters may confer temporary or long-term transcriptional inhibition, resulting in reduced amenability to selection and gene expression.

In plants, cytosine methylation occurs at CG, CHG, and CHH sites (where H = A, C, or T). In *Arabidopsis*, *de novo* methylation at CG, CHG, and CHH sites can be established by the DOMAINS REARRANGED METHYLTRANSFERASE 2 (DRM2) (Cao and Jacobsen, 2002). Maintenance of CG methylation is carried out by METHYLTRANSFERASE 1 (MET1) (Saze et al., 2003). *De novo* methylation and maintenance in the CHG context is carried out by the co-ordinated action of DRM2 and CHROMOMETHYLASE 3 (CMT3) (Cao et al., 2003). Methylation maintenance in the CHH context is either re-established by DRM2 or CMT3 via an RNA directed DNA methylation (RdDM) trigger or is carried out independently of the RdDM trigger by CMT2 within transposons (Chan et al., 2005; Law and Jacobsen, 2010; Matzke and Mosher, 2014).

Transgenes can be silenced via post-transcriptional gene silencing (PTGS) and via transcriptional gene silencing (TGS). In PTGS, the targeted transcript is degraded by RNA interference (RNAi) machinery (Matzke and Mosher, 2014) and in many cases subsequently invokes TGS (Sijen et al., 2001; Nocarova et al., 2010). TGS inhibits the transcription of target genes and is strongly associated with promoter methylation. Briefly, the pathway in plants is as follows: the plant specific RNA Pol IV first produces the ssRNA from the transgene. Through the action of RDR6, this ssRNA is made into dsRNA. The ribonuclease III activity of DICER-LIKE 3 (DCL3) then processes the dsRNA into 24 nt siRNAs which are loaded into ARGONAUTE 4 or 6 (AGO4/6). Interaction in a sequence specific manner between the loaded AGO4/6, ssRNA RNA Pol V transcripts and DRM2 results in *de novo* methylation of the transgenic DNA template (Matzke and Mosher, 2014).

Including certain intron sequences in transgene constructs, in or near the 5′ UTR, is known to improve expression, termed intron-mediated enhancement (IME) (Mascarenhas et al., 1990). IME is thought to be due to an increase in RNA Pol II binding, the recruitment of transcription factors, or a combination of both (Morello and Breviario, 2008; Laxa et al., 2016). The orientation of the intron, position within the transcribed region, proximity to the transcription start site (TSS) and specific sequence motifs may all contribute to IME (Rose et al., 2008; Laxa, 2017). But little is known about the role promoter methylation may play in the phenomena.

Here, we investigate the methylation of T-DNA in the early stages following transient *Agrobacterium* infiltration of *Nicotiana benthamiana* leaves. The status of two transgene promoters, CaMV-35S and AtEF1α-A4 (with and without its 5′ UTR intron) was followed over the first few days and weeks post-infiltration.

## Materials and Methods

### Constructs Used

A 35S-eGFP vector was generated by cloning a cassette containing the 35S promoter followed by eGFP and the OCS terminator into pORE 03 (Coutu et al., 2007) via the HindIII and EcoRI sites (Supplementary Figure S1). A hairpin construct, pSSU-eGFP-F-hp targeting fragment ‘F’ of eGFP was used to enhance PTGS in the time course (McHale et al., 2013).

The 35S promoter was excised via the HindIII and XhoI sites from the 35S-eGFP vector to maintain the same vector backbone. To generate the 5′ UTR intron containing construct, the 1417 nt sequence upstream of the *AtEF1α-A4* (AT5G60390.1) gene to the TSS was isolated using the primers EF1a-4_Prom_GA_F and EF1a-4_Prom_+Int_GA_R. To generate the 5′ UTR intronless version, a reverse primer EF1a-4_Prom_-Int+CT_GA_R was used in combination with EF1a-4_Prom_GA_F. Polymerase chain reaction (PCR) fragments were introduced into the digested vector via Gibson Assembly (NEB) and transformed into electrocompetent DH5α using standard molecular techniques. Primer sequences are presented in Supplementary Table S1.

### Transformations and Preparation of *Agrobacterium tumefaciens* GV3101 (MP90)

Sequence verified plasmids were transformed into electrocompetent *Agrobacterium tumefaciens* GV3101 (MP90) using standard molecular techniques (Wise et al., 2006). A single colony was then grown in 5 mL of LB culture supplemented with the appropriate antibiotic selection for 16 h at 28°C with shaking at 180 rpm. Then 100 μM acetosyringone (PhytoTechnology Laboratories) was added and cells returned to incubate at 28°C with shaking at 180 rpm for 90 min. Cells were then washed twice in 5 mL of infiltration buffer (10 mM MgCl_2_, 10 mM MES hydrate, pH 5.7) supplemented with 100 μM acetosyringone. Cultures were then adjusted to an OD_600_ of 0.2 for agro-infiltrations.

### Agro-Infiltrations

For infiltrations, the *Agrobacterium* solutions were mixed in equal volume ratios prior to infiltration. The mixtures were agro-infiltrated via a 1 mL syringe onto the underside of *N. benthamiana* LAB (Bally et al., 2015) leaves at 4 weeks of age. The growth conditions are as described in Philips et al. (2017). For the sequencing platform comparison experiment, a single leaf was infiltrated. For the base-pair resolution experiment, three plants with three leaves each were infiltrated per time point. The plants were allowed to grow for a further 21 days for 35S-eGFP experiments and 7 days for AtEF1α-A4 ± 5′ UTR intron experiments. These infiltrated leaves were collected at various time points, imaged, uniform patches cut and stored at -80°C for DNA and RNA extractions.

### Imaging

Infiltrated leaves were visualized using a Dark Reader Hand Lamp HL32T (Clare Chemical). Images were captured using a Canon EOS 550D DLSR camera using an EF-S 60 mm lens attached with a Hoya HMC O(G) amber filter blocking blue light transmission (Supplementary Figure S2). Captured images were processed using ImageJ v1.50i to determine the mean gray value indicative of eGFP fluorescence intensity as described in Wydro et al. (2006).

### DNA Extractions, Precipitation, and Bisulfite Conversion

Approximately 100–200 mg of infiltrated leaf were ground under liquid nitrogen and used in DNA extractions. The NucleoSpin Plant II (Macherey-Nagel) and the DNeasy Plant Mini Kit (Qiagen) extraction kit were used for the sequencing platform comparison and the final experiments, respectively. DNA was precipitated by ^1^/_10_ v/v sodium acetate and 2.5x v/v ethanol and resuspend in elution buffer to a concentration of ∼110 ng/μL. Between 1250 and 1750 ng of this DNA was converted using the EpiTect Fast DNA Bisulfite Kit (Qiagen) with the 60°C incubation extended to 20 min to achieve maximum conversion. The bisulfite converted DNA was then diluted with molecular grade water to ∼5 ng/μL and used as a template for PCR.

### Bisulfite PCR

#### Sanger Sequencing

Polymerase chain reaction amplifications were carried out as follows; an initial denaturation step at 95°C for 3 min, followed 40 cycles comprising of 95°C for 15 s, 55°C for 15 s, and 72°C for 15 s, followed by a final extension for 1 min at 72°C. Reactions contained 1x KAPA2G Robust HotStart ReadyMix (KAPA), 0.5 μM each of dBS F4.1 and dBS R7.1 primers (Supplementary Table S1), 4 μL (∼20 ng) of diluted bisulfite converted DNA as template and the volume made up to 20 μL with molecular grade water.

#### For Ion Torrent

Touchdown PCR amplifications were carried out as follows; an initial denaturation step at 95°C for 3 min, followed by 10 touchdown cycles comprising of 95°C for 15 s, 70°C for 15 s, and 72°C for 15 s, with the annealing temperature decreasing by 1°C per cycle, followed by 29 cycles comprising of 95°C for 15 s, 55°C for 15 s, and 72°C for 15 s, followed by a final extension for 1 min at 72°C. Reactions contained 1x KAPA2G Robust HotStart ReadyMix (KAPA), 0.5 μM each of forward and reverse Ion Torrent fusion primers with adapters (Supplementary Table S1), 3 mM MgCl_2_, 4.5 μL (∼22.5 ng) of diluted bisulfite converted DNA as template and the volume made up to 20 μL with molecular grade water.

#### Extension Temperature Optimization Using Illumina MiSeq

Touchdown gradient PCR amplifications were carried out as follows; an initial denaturation step at 95°C for 3 min, followed by 10 touchdown cycles comprising of 95°C for 20 s, 67°C for 20 s, and 66–75°C for 45 s, with the annealing temperature decreasing by 1°C per cycle, followed by 29 cycles comprising of 95°C for 20 s, 54°C for 20 s, and 66–75°C for 45 s, followed by a final extension for 2 min at 66–75°C. To reduce variability, reactions cycled at the same time. Column 4 within the Mastercycler nexus GSX1 (Eppendorf) was selected for 67.5°C (actual 67.6°C) and column 8 was selected for 72°C (actual 72.2°C).

NEB reactions contained 1x EpiMark Hot Start *Taq* Reaction Buffer (NEB), 300 μM of each dNTP (KAPA), 0.5 μM each of dBS F4.1 Illumina and dBS R7.1 Illumina fusion primers (Supplementary Table S1), 3.3 mM of MgCl_2_, 6.25 μL (∼31.25 ng) of diluted bisulfite converted DNA as template, 1.25 U EpiMark Hot Start *Taq* DNA polymerase (NEB), and the volume made up to 25 μL with molecular grade water.

KAPA reactions contained 1x KAPA2G Robust HotStart ReadyMix (KAPA), 0.5 μM each of dBS F4.1 Illumina and dBS R7.1 Illumina fusion primers (Supplementary Table S1), 3 mM MgCl_2_, 6.25 μL (∼31.25 ng) of diluted bisulfite converted DNA as template and the volume made up to 25 μL with molecular grade water.

#### Base-Pair Resolution Experiment Using Illumina MiSeq

Touchdown PCR amplifications and cycling using EpiMark Hot Start *Taq* DNA polymerase (NEB) was carried out as described for the extension temperature optimization experiment with an extension temperature of 67.5°C and scaled down to a volume of 15 μL.

### NGS Library Preparation

Polymerase chain reaction amplicons were submitted to the Central Analytical Research Facility (CARF) at QUT. For Ion Torrent, the PCR amplicons were purified, pooled in equimolar amounts to prepare the library and then diluted to ∼26 pM. Sequencing was carried out on the Ion 318 Chip v2 with the Ion PGM Hi-Q View Sequencing Kit (400 base reads). For Illumina MiSeq tagmentation, the amplicons were treated with the Nextera transposome and adapters added using the Nextera XT index kit v2. The purified amplicons were then pooled in equimolar amounts and diluted to a concentration of 2 nM. The library was denatured, diluted to ∼6 pM with 10% PhiX spike-in, loaded on a MiSeq flow cell and sequencing carried out on the Illumina MiSeq with MiSeq v3 reagent chemistry. For the Illumina MiSeq fusion approach, the Nextera XT indices v2 were added in a secondary PCR. The library was then treated and sequenced as described for the tagmentation procedure with the exception of 25% PhiX spike-in. All procedures were followed as per manufacturer’s instructions. These data are accessible at www.benthgenome.com.

### Methylation Analysis

#### For Ion Torrent PGM

Ion Torrent reads were processed using Trimmomatic v0.32.2 (Bolger et al., 2014). The parameters included sliding window trimming of four bases and an average quality Phred score of 12 (2 and 3 dpi) and Phred score of 22 for all other time points. Reads shorter than 395 bp were excluded to ensure only full length reads were analyzed. Trimmed reads were aligned and columns containing more than 90% gaps were stripped using Geneious 11.0.2 (Biomatters, Ltd.) Methylation analysis was performed on these processed reads using the web-based tool Kismeth (Gruntman et al., 2008) and the output used to tabulate methylation percentages in all cytosine contexts.

#### For Illumina MiSeq – Sequencing Platform Comparison Experiment

Pair-end MiSeq reads were merged using PEAR v0.9.6.0 (Zhang et al., 2014). Residual Illumina adapter sequences were removed using Cutadapt v1.6 (Martin, 2011). These reads were then processed using Trimmomatic as described for Ion Torrent but with an average Phred score of 30. Due to the tagmentation approach full length reads of 418 bp were not possible so all reads below 250 bp were excluded. For Illumina fusion, reads below 395 bp were excluded. Processed reads were analyzed as per the Ion Torrent procedure.

#### For Illumina MiSeq – Base-Pair Resolution Experiment

MiSeq reads were merged as described earlier. Reads were then processed within Geneious 11.0.2 (Biomatters, Ltd.) using the BBDuk plugin with the following settings: Phred score of 25 and minimum read length of 395 bp for 35S-eGFP time courses and Phred score of 20 and minimum read length of 525 bp for AtEF1α-A4 ± 5′ UTR time courses. The processed reads were then aligned, columns stripped and analyzed as per the Ion Torrent procedure. The number of total raw reads generated is presented in Supplementary Table S2.

### RNA Extraction, cDNA Synthesis, and qRT-PCR

RNA from each replicate was extracted, reverse transcribed by PrimeScript RT reagent Kit (Perfect Real Time, TaKaRa Bio, Inc.) and processed as described in Tashiro et al. (2016). Assays were designed and executed according to MIQE guidelines (Bustin et al., 2009). *PP2A* and *L23* as described in Liu et al. (2012) were used as normalization factors. Relative expression assays were run on the CFX384 Touch Real-Time PCR detection system (BioRad) and the data analyzed with the supplied software. Final reaction volumes were 10 μL that consisted of: 4 μL of diluted cDNA, 0.2 μM each of forward and reverse primer (Supplementary Table S1), and 1x SYBR Premix Ex Taq II (Tli RNase H Plus, TaKaRa Bio, Inc.). All liquid handling steps were performed using an Eppendorf epMotion 5075. PCR efficiency was determined via a standard curve of linearized plasmid as described by Tashiro et al. (2016). The samples, standard curve and no-template controls were repeated in technical triplicates.

### Statistical Analysis

General linear model (two-way ANOVA) using combined CG, CHG, and CHH methylation was compared at each time point between the time courses. ANOVA on the level of methylation intensity was performed on the data presented in Figure 6. All tests were performed using Minitab 17 (Minitab, Inc.).

## Results

### eGFP Fluorescence and Transcript Decrease Over Time

**FIGURE 1:**
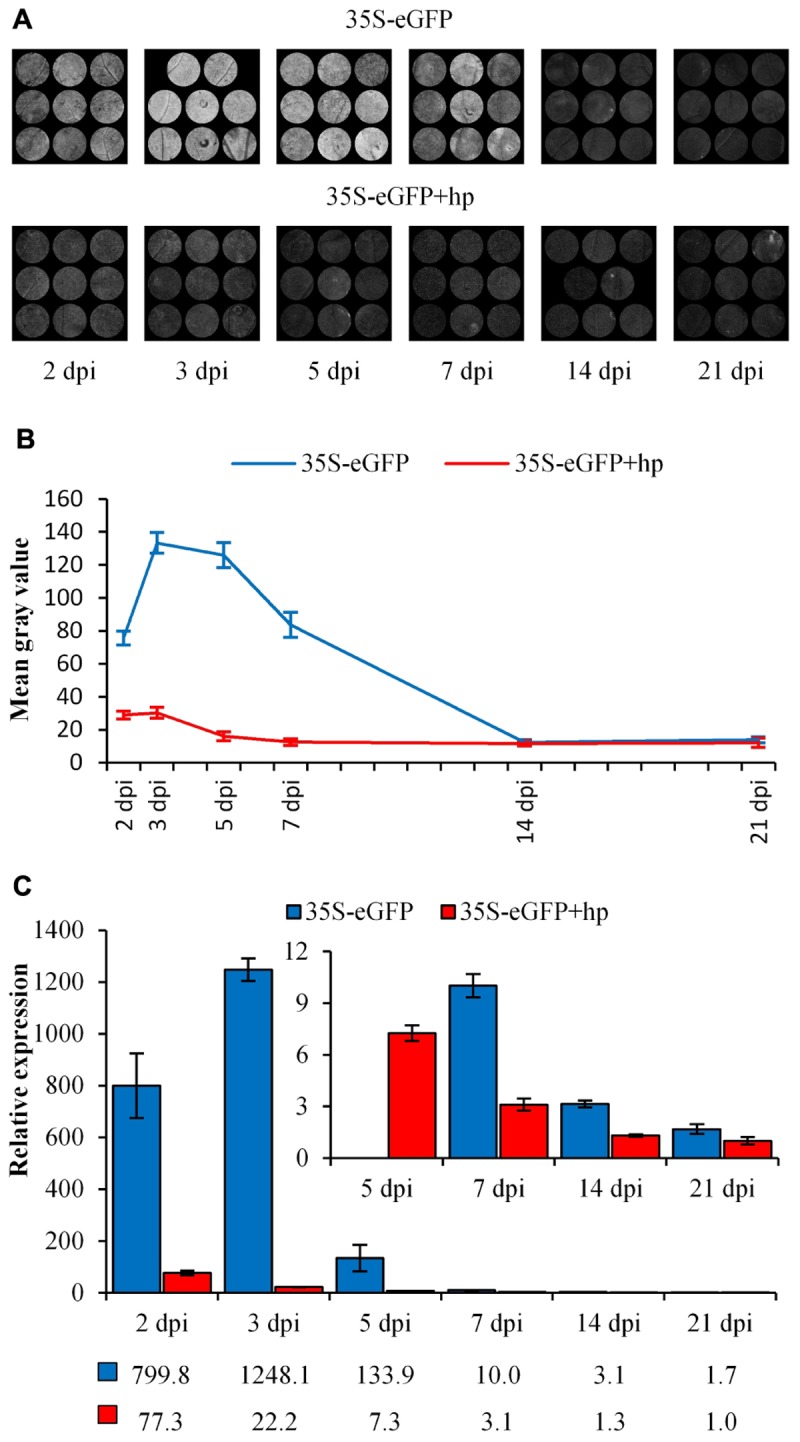
eGFP fluorescence and relative transcript abundance in *Nicotiana benthamiana* leaves over 21 days post-infiltration. Time courses are either 35S-eGFP or co-infiltration with a hairpin (35S-eGFP+hp), targeting *eGFP* to induce PTGS. **(A)** Processed images of infiltrated leaf spots subjected to ImageJ analysis. **(B)** eGFP fluorescence indicated by mean gray values, *n* = 8 or 9, means ± SEM. **(C)** Relative transcript abundance of *eGFP*, with *PP2A* and *L23* used as normalization factors. The expression of the 21 dpi time point in the 35S-eGFP+hp treatment was set to 1. Relative transcript abundances are presented below the graph, *n* = 3, means ± SEM.

The levels of transient eGFP fluorescence and transcript was measured over a 21-day time course after infiltrating *N. benthamiana* leaves with a 35S-eGFP construct. As the loss of fluorescence and transcript in transiently infiltrated plants has been implicated with PTGS (Voinnet et al., 2003), a second experiment where a hpRNA construct targeting eGFP itself was co-infiltrated to enhance PTGS. The enhanced PTGS is due to the production of dsRNA which are processed by the DICER-LIKE (DCL) proteins to generate the siRNAs needed for gene silencing (Helliwell and Waterhouse, 2003). In both time courses, the accumulation and subsequent reduction of eGFP fluorescence and relative transcript abundance is observed (Figure 1). Following infiltration, eGFP fluorescence and relative transcript abundance peaked at 3 dpi and fluorescence was detectable over the first week in the 35S-eGFP time course. In comparison, a reduced eGFP fluorescence and transcript abundance was observed in the 35S-eGFP+hp time course and fluorescence was undetectable at an earlier time point of 5 dpi (Figure 1).

#### Sequencing Platform Comparisons

We then assessed DNA methylation of the T-DNA to determine if this correlated with the loss of eGFP fluorescence and transcript observed in Figure 1. The 14 dpi sample from the 35S-eGFP+hp time course was selected and tested on different sequencing platforms to determine which would most appropriately obtain DNA methylation from a heterogeneous population of T-DNA.

**FIGURE 2:**
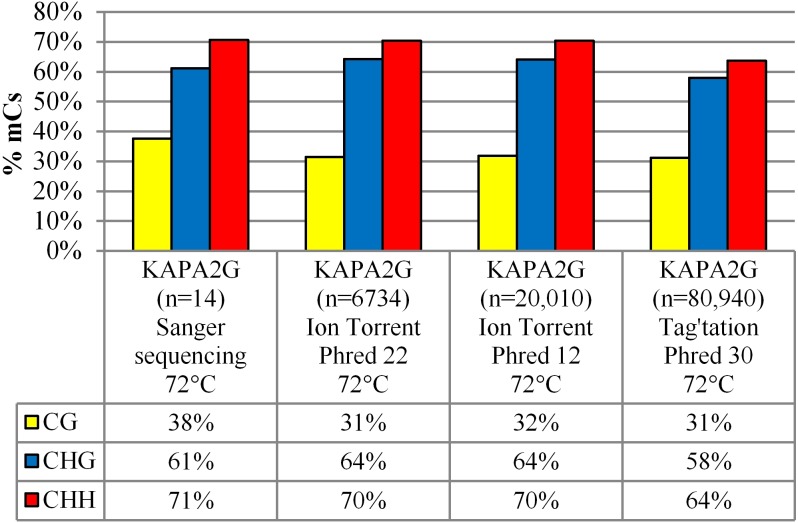
Sequencing platform comparison between Sanger sequencing, Ion Torrent PGM and Illumina MiSeq using the tagamntation approach. The percentage of methylated cytosines (% mCs) is presented for the 14 dpi 35S-eGFP+hp sample, n = number of reads used from one infiltrated leaf to build the profile after quality and length filtering.

The amplicon produced using bisulfite-treated DNA and the degenerate primers (dBS F4.1 and dBS R7.1, Supplementary Table S1) was cloned into a TA vector and 14 colonies representing individual T-DNAs were analyzed using Sanger sequencing. This produced an overall 38% (CG), 61% (CHG), and 71% (CHH) cytosine methylation profile (Figure 2).

Cytosine methylation was then assessed using the Ion Torrent PGM sequencer with the same bisulfite-treated DNA amplified with degenerate Ion Torrent fusion primers (Supplementary Table S1). Sequencing the amplicon produced a total of 457,552 individual sequences of various lengths. These sequences were filtered for a minimum length of 395 bp which represents the length comprising of all the cytosines in the amplicon. To assess whether the methylation profile was affected by altering filtering quality threshold, two different Phred quality scores of 12 and 22 were selected, representing a base call accuracy of 93.67 and 99.37%, respectively. The lower Phred score allowed a higher number of T-DNA amplicon sequences to be analyzed (20,010 as opposed to 6734) but did not affect the deduced cytosine methylation profile of 32% (CG), 64% (CHG), and 70% (CHH) for Phred 12 and marginally lowered the CG context to 31% for Phred 22 with the CHG and CHH contexts remaining unchanged (Figure 2 and Table 1).

**Table 1:** Comparisons amongst the sequencing platforms and parameters used.

Amplification		Sequencing	Criteria		Reads			Methylation		
				
Enzyme	Extension temperature (°C)	Platform – Index	Min. length (bp)	Q score	Total reads	Filtered reads	% Used reads	CG (%)	CHG (%)	CHH (%)
KAPA2G Robust	72	Sanger Sequencing	418	N/A	14	14	100	38	61	71
KAPA2G Robust	72	Ion Torrent – Fusion	395	Q22	457,552	6734	1.5	31	64	70
KAPA2G Robust	72	Ion Torrent – Fusion	395	Q12	457,552	20,010	4.4	32	64	70
KAPA2G Robust	72	MiSeq – Tagmentation – Nextera XT v2	250	Q30	374,865	80,940	21.6	31	58	64
KAPA2G Robust	72.2	MiSeq – Fusion – Nextera XT v2	395	Q30	19,942	15,027	75.4	31	62	70
KAPA2G Robust	67.6	MiSeq – Fusion – Nextera XT v2	395	Q30	22,398	16,685	74.5	17	32	34
EpiMark Hot Start (NEB)	67.6	MiSeq – Fusion – Nextera XT v2	395	Q30	37,287	25,716	69.0	15	30	33


*Amplification with extension temperatures at either 72.2, 72, or 67.6°C was carried out with either KAPA2G Robust or EpiMark Hot Start (NEB). The criteria used to filter reads and the percentage of reads used in the final analysis is presented along with the methylation profile.*

To improve the number of reads after filtering the Illumina MiSeq sequencing platform was then used with the same bisulfite-treated DNA amplified with degenerate primers (dBS F4.1 and dBS R7.1, Supplementary Table S1). A tagmentation approach was then used for adapter and index addition to differentiate multiple samples. A reduced 31% (CG), 58% (CHG), and 64% (CHH) cytosine methylation profile was observed using this approach compared to the profiles attained by both Ion Torrent and Sanger sequencing (Figure 2).

### Comparisons Between Ion Torrent and Illumina MiSeq

**FIGURE 3:**
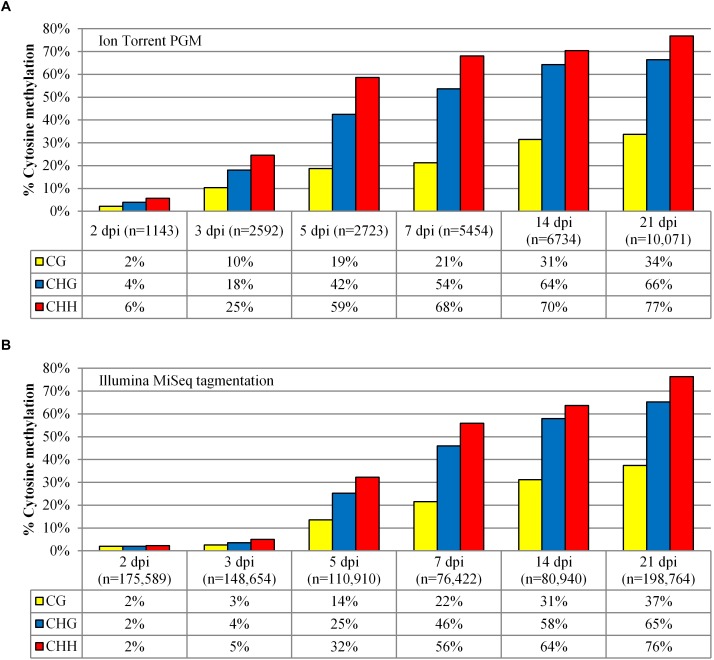
Methylation analysis comparison of the 35S-eGFP+hp time course experiment. **(A)** Methylation analysis was performed after the samples were sequenced using the Ion Torrent. A minimum length of 395 bp was selected with Phred scores of Q12 for 2 and 3 dpi, and Q22 for the remaining time points. **(B)** Methylation analysis on the same samples sequenced using the Illumina MiSeq tagmentation approach. A minimum length of 250 bp was selected with Phred scores of Q30 for all samples. n = number of reads used from one infiltrated leaf to build the profile after quality and length filtering.

As more T-DNA amplicon sequence reads are able to be cost-effectively assessed by NGS rather than Sanger sequencing, a time course of the methylation profile compared only the Ion Torrent and Illumina MiSeq sequencing platforms. A similar trend in T-DNA methylation over time was observed using both NGS platforms. There was a greater increase in CG, CHG, and CHH methylation from 3 dpi to 5 dpi followed by a steady increase in accumulation to 21 dpi (Figure 3). However, for Ion Torrent data, the samples at the earlier time points (prior to 5 dpi inclusive) showed a comparatively higher level of methylation. Furthermore, the Ion Torrent time course experiment showed a correlation between the number of reads and the degree of methylation. For example, there were fewer reads with low methylated profiles in early time points compared to later time points which had a higher number of reads and increased methylation (Figure 3). There was also a high level of truncated reads on the Ion Torrent platform in these earlier time points, for example, the 2 dpi sample had 3.8% of reads over 395 bp, whereas the 14 dpi sample had 18.8% of reads over 395 bp prior to any Phred score filtering (Supplementary Table S3).

A limitation of the tagmentation approach was the reduction in full length amplicon reads due to the fragmentation required for index addition to the PCR amplicon. Following quality score filtering of Q30 and selecting amplicons larger than 250 bp, only 21.6% of the generated reads were available for cytosine methylation profile analysis (Table 1). Thus to increase the number of full length reads available for analysis, the same bisulfite-treated DNA was amplified with the degenerate bisulfite primers with adapters added as overhangs (fusion approach, Supplementary Table S1). Following quality score filtering of Q30 and selecting full length amplicon reads, 75.4% of the generated reads were now available for cytosine methylation profile analysis (Table 1). A similar 31% (CG), but higher 62% (CHG) and 70% (CHH) cytosine methylation profile was observed for the fusion approach compared to the tagmentation approach (Figure 2).

In summary, NGS platforms produce several 1000 reads that are able to be analyzed for their methylation status. The protocol allowed for barcoding and multiplexing which is otherwise laborious when sequencing individual colonies via Sanger sequencing. Having a greater sequencing depth is particularly important when a heterogeneous pool of differentially methylated T-DNAs may exist, such as that in transiently infiltrated leaf tissue. Further, we have found the Illumina fusion based approach to be most suitable and this was used in subsequent experiments.

### PCR Extension Temperature Comparison

**FIGURE 4:**
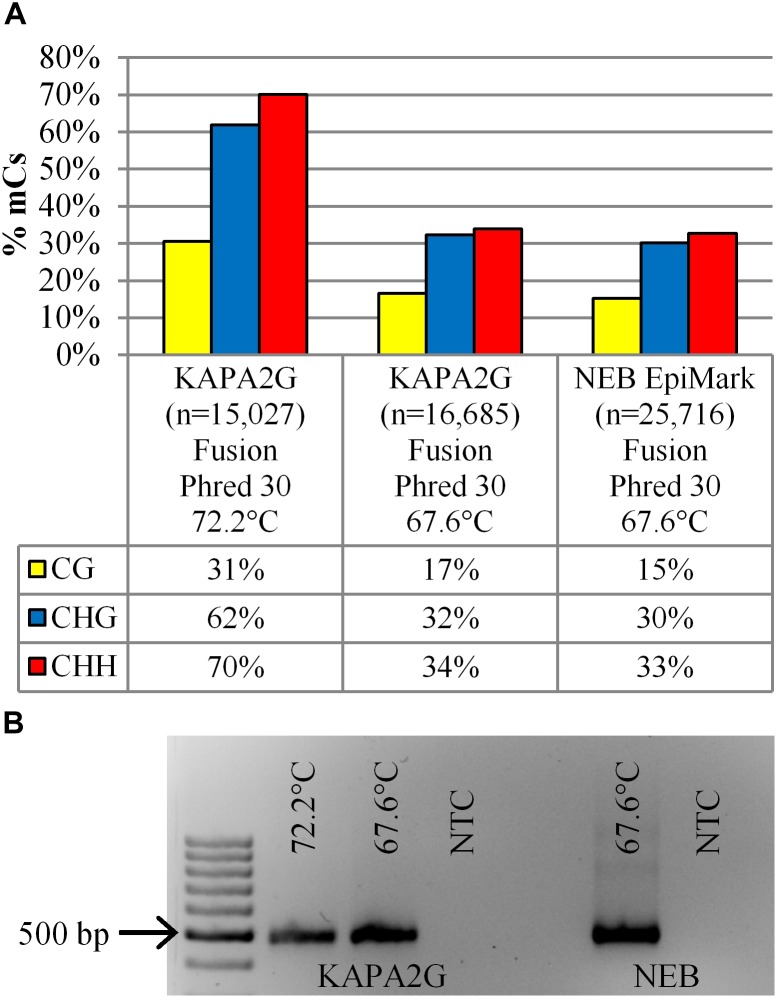
**(A)** The percentage of methylated cytosines (% mCs) is presented for the 14 dpi 35S-eGFP+hp sample sequenced on the Illumina MiSeq with the fusion protocol. Comparisons are amongst the DNA polymerases (KAPA2G and NEB EpiMark) and extension temperature used during the bisulfite PCR; n = number of reads used from one infiltrated leaf to build the profile after quality and length filtering. **(B)** Gel electrophoresis showing amplification intensity of the same sample used to generate the methylation profile in **(A)**; NTC, no template control. 100 bp DNA Ladder (GeneRuler).

To assess whether the PCR extension temperature affected the inferred cytosine methylation profile, the extension temperature of the bisulfite-treated DNA in the KAPA2G Robust reaction was reduced from 72°C (72.2°C actual) to 67.5°C (67.6°C actual) and the amplicons visualized on an agarose gel (Figure 4B) followed by analysis using the Illumina MiSeq fusion approach. Interestingly, irrespective of the polymerase (KAPA2G Robust or NEB EpiMark), using an extension temperature of 67.6°C resulted in a twofold reduction in the methylation profile of all cytosine contexts when compared to the methylation profile of the KAPA2G Robust reaction with PCR extension taking place at 72.2°C (Figure 4A).

Taken together, the results from the sequencing platform comparison experiment provide confidence that the developed assay was reproducible and demonstrated methylation accumulation over time. The time course experiment was then repeated at base-pair resolution with biological replication with sequencing taking place on the Illumina MiSeq using the fusion approach. For this experiment, amplification from bisulfite converted DNA was carried out using the EpiMark Hot Start *Taq* polymerase (NEB) with PCR extension taking place at 67.5°C.

### Rapid T-DNA Methylation Following Agro-Infiltration

**FIGURE 5:**
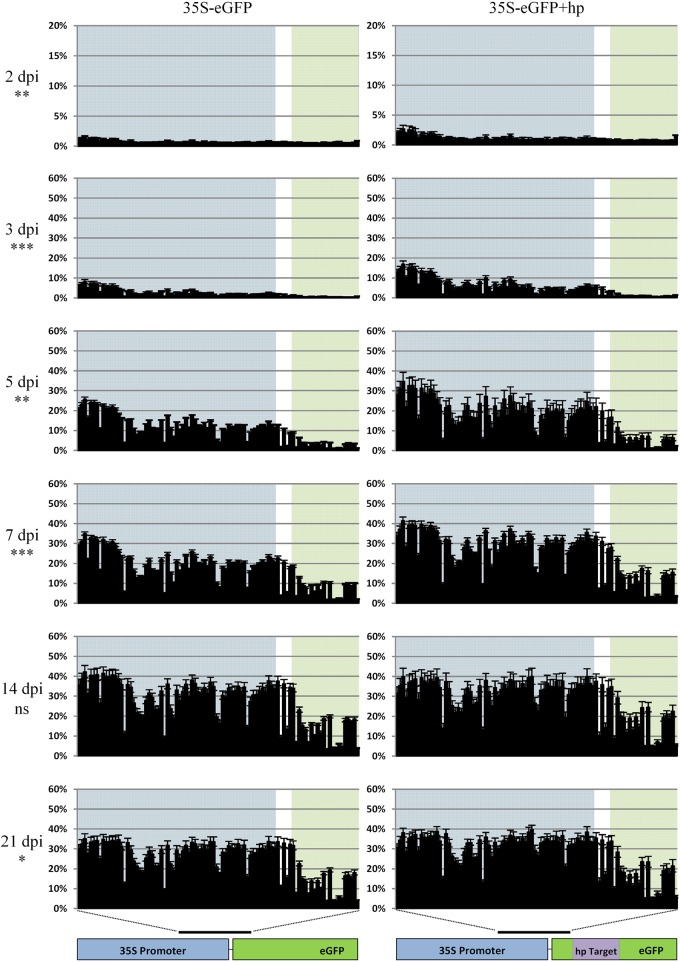
Methylation analysis comparison in the time course of the 35S-eGFP transgene without a hairpin (left panel) or with a hairpin (right panel) targeting the eGFP transgene (shown in purple). Percentage cytosine methylation is presented on the y-axis and individual cytosine positions in the 418 bp region indicated by the black bar on the x-axis. Background highlights: blue: promoter, white: 5′ UTR and green: coding sequence (CDS). Sequencing was carried out on the Illumina MiSeq with the fusion protocol, n = three biological replicates. General linear model (two-way ANOVA) followed by the Tukey test was used to compare all cytosine contexts between the two treatments at each time point. Statistically significant differences, ^ns^ not significant, ^∗^*P* < 0.05, ^∗∗^*P* < 0.01, ^∗∗∗^*P* < 0.001.

Methylation analysis in base-pair resolution of the 3′ region of the 35S promoter and part of the 5′ region of eGFP was carried out over a 3 week time course with collections at 2, 3, 5, 7, 14, and 21 dpi with biological replication (Figure 5). In these experiments, the methylation increased over the time course as previously observed in the sequence platform comparison experiments (Figure 3) and was detected in as little as 2 dpi. In the first week (2, 3, 5, and 7 dpi) there is a higher combined cytosine methylation profile when the hpRNA was co-infiltrated with 35S-eGFP (*P* < 0.01). At 14 dpi although higher, the methylation profile of the hpRNA containing time course was not significantly different to the time course without the hpRNA (*P* > 0.05). However, a significant increase in the combined cytosine methylation profile was evident at the 21 dpi time point in the hpRNA containing time course (*P* < 0.05). Overall, T-DNAs are rapidly methylated once transiently infiltrated into *N. benthamiana* leaf tissue. The cytosine context comparison at each time point and the methylation of individual bases is presented in Supplementary Figures S3, S4.

### T-DNA Methylation From a Heterogeneous Population

A range of T-DNAs are likely to exist following agro-infiltration into *N. benthamiana* leaves. These include: (1) un-nicked T-DNA residing on the Ti plasmid still within the *Agrobacterium*, (2) nicked single-stranded T-DNA (ssT-DNA) either within the *Agrobacterium* or transferred to the plant cell, (3) linear double-stranded dsT-DNA (dsT-DNA) within the plant cell, (4) circularized and unintegrated dsT-DNA within the plant cell, as described by Singer K. et al. (2012) and (5) integrated T-DNA within the plant genome. As we have isolated total genomic DNA from the agro-infiltrated patch, the deduced methylation profiles will contain a contribution from some DNA still within the *Agrobacterium*. To determine the extent of *Agrobacterium* T-DNA contribution, individual T-DNAs amplicons at each time points in the 35S-eGFP+hp experiment were analyzed for methylation intensity. The amplicon generated using the dBS F4.1 and dBS R7.1 Illumina fusion primers (Supplementary Table S1) contains 92 cytosine sites. After bisulfite conversion and PCR, individual T-DNA reads were grouped based on their number of cytosines into low (0, 1, and 2), medium (3–30 and 31–60), and high (61–92), which is indicative of the intensity of cytosine methylation. Individual T-DNA reads with zero to low levels of cytosines most likely originate from the T-DNAs either within the *Agrobacterium* or those being transferred into the plant. Analysis from the hpRNA-containing time course was used as the hairpin will expedite gene silencing such that once in the plant, methylation of the T-DNAs is more likely to occur. Therefore, this time course rather than 35S-eGFP by itself will better serve to indicate the maximum level of *Agrobacterium*-retained T-DNA in the heterogeneous population.

**FIGURE 6:**
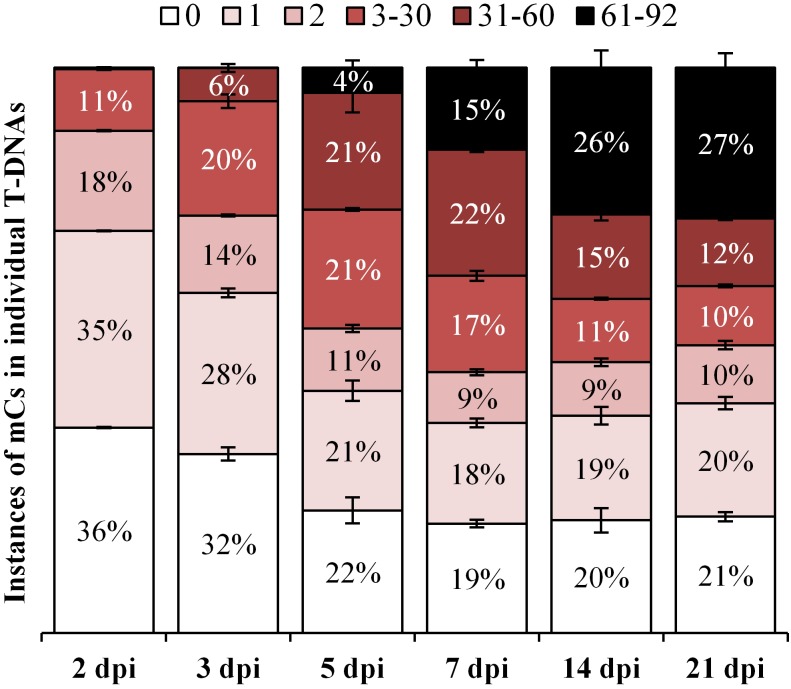
Frequency of methylation intensity in individual T-DNAs from a heterogeneous population over the 35S-eGFP+hp time course. The fully methylated PCR product amplified by the dBS F4.1 and dBS R7.1 Illumina fusion primers (Supplementary Table S1) contains 92 cytosine sites. Individual T-DNA reads are grouped based on their intensity by the number of methylated cytosines (mCs) into low (0, 1, and 2), medium (3–30 and 31–60) and high (61–92); *n* = 3 biological replicates, means ± SEM.

During the early stages following agro-infiltration, 36 and 32% of all T-DNA reads had zero methylation at 2- and 3- dpi, respectively. Interestingly, at the later time points (5, 7, 14, and 21 dpi) a consistent level of 22, 19, 20, and 21% of zero methylation is observed, respectively (*P* > 0.05, Figure 6) and this probably reflects the contribution to the analysis from T-DNA within *Agrobacterium*. Importantly, at these same time points, the percentage of highly methylated reads (containing between 61 and 92 methylated cytosines) steadily increases from 4 to 27% (Figure 6). This demonstrates a continuing methyltransferase activity within the plant rather than a loss of T-DNAs still retained by the *Agrobacterium*.

### Inclusion of AtEF1α-A4 5′ UTR Intron Results in IME and Decreases Promoter Methylation

**FIGURE 7:**
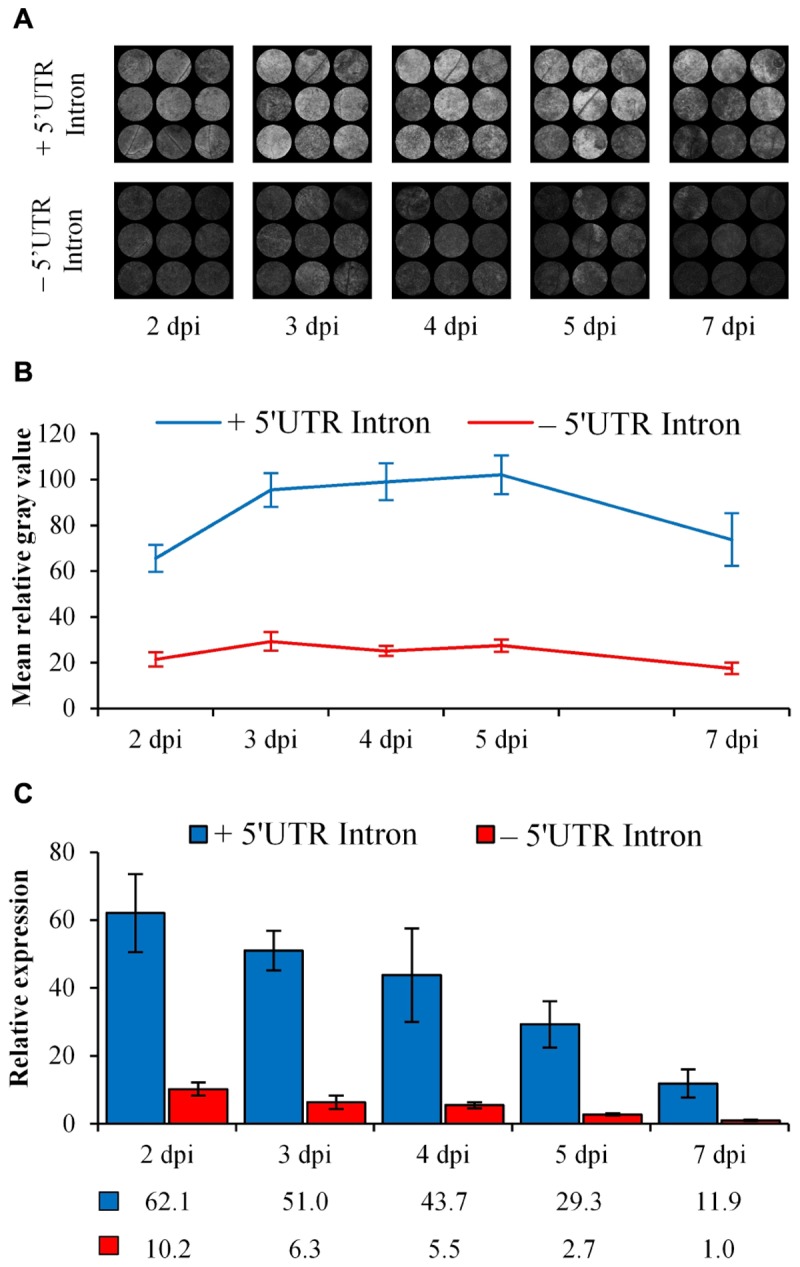
Comparison of eGFP fluorescence and relative expression when regulated by either the AtEF1α-A4 promoter with (+) or without (–) the 5′ UTR intron over 7 days in *N. benthamiana* leaves. **(A)** Processed images of spots subjected to ImageJ analysis. **(B)** eGFP fluorescence indicated by mean gray values, *n* = 9, means ± SEM. **(C)** Relative transcript abundance of *eGFP*, with *PP2A* and *L23* used as normalization factors. The expression of the 7 dpi time point in the –5′ UTR treatment was set to 1. Relative transcript abundances are presented below the graph, *n* = 3, means ± SEM.

Intron-mediated enhancement was seen with increased eGFP fluorescence and relative transcript abundance when under the control of the AtEF1α-A4 promoter containing the 5′ UTR intron compared to the intronless construct (Figure 7). The mean relative gray value, indicative of eGFP fluorescence, was threefold higher at the first time point (2 dpi) with the intron-containing construct (Figure 7B) and increased to nearly fourfold by 4 dpi (Figure 7B). At 7 dpi, there was a decrease in eGFP fluorescence as seen with the 35S-eGFP time course experiment (Figure 1). An intron-spanning RT-PCR confirmed that the 5′ UTR was correctly processed (Supplementary Figure S5). qRT-PCR was performed to test whether an increase in eGFP fluorescence was due to an increase in mRNA transcript abundance. Comparatively, there was a higher *eGFP* transcript abundance in the 5′ UTR intron containing construct at all time points tested (Figure 7C).

**FIGURE 8:**
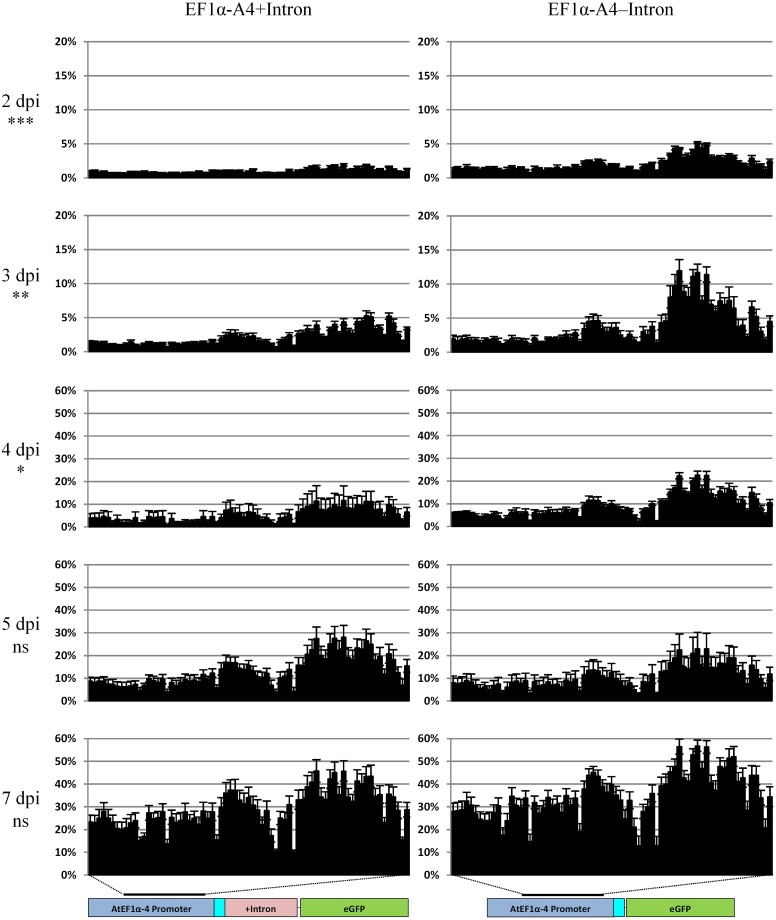
Methylation analysis comparison in the time course of the AtEF1α-A4 promoter with (left panel) or without the 5′ UTR intron (right panel) regulating the expression of *eGFP*. Percentage cytosine methylation is presented on the y-axis and individual cytosine positions in the 546 bp region indicated by the black bar on the x-axis. Sequencing was carried out on the Illumina MiSeq with the fusion protocol, n = three biological replicates. General linear model (two-way ANOVA) followed by the Tukey test was used to compare all cytosine contexts between the two treatments at each time point. Statistically significant differences, ^ns^not significant, ^∗^*P* < 0.05, ^∗∗^*P* < 0.01, ^∗∗∗^*P* < 0.001. Note the difference in the scale used for the y-axis at 2 and 3 dpi compared to 4, 5, and 7 dpi.

To test whether IME correlated with a decrease in methylation, analysis of the promoter involving biological replication was carried out over a 1 week time course with collections at 2, 3, 4, 5, and 7 dpi (Figure 8). As observed in our 35S-eGFP time course experiments (Figure 5), T-DNA methylation of the promoter was detected after agro-infiltration into the plant. There was a significant difference in the combined cytosine contexts (*P* < 0.05) between the 5′ UTR intron-containing and intronless promoters at the 2, 3, and 4 dpi time points. This indicates that the inclusion of the 5′ UTR intron, in addition to IME, causes a reduction in the level of promoter methylation. At 5 and 7 dpi, there was no significant difference (*P* > 0.05) in the methylation profile of the promoters between these two treatments. The cytosine context comparison at each time point and the methylation of individual bases is presented in Supplementary Figures S6, S7.

## Discussion

### Sequencing Platform Comparisons

Three different sequencing platforms were compared in this study with the same bisulfite-treated DNA sample used from the infiltrated *N. benthamiana* leaves. Sequencing was carried out at different times over the course of the comparison experiment with the sample stored at -80°C to ensure viability. The methylation profile, regardless of the platform used, was comparable when PCR extension took place at 72°C, indicating the reproducibility of the bisulfite-treated DNA sample on different platforms (Figure 2). The total number of reads in each experiment varied as this was dependent on the total number of unique samples pooled in the sequencing run.

Degenerate bisulfite primers targeting the T-DNA, without any overhangs necessary for NGS indexing, were first utilized and the methylation profile assessed via Sanger sequencing. The results confirmed that the T-DNA is methylated in the early stages following *Agrobacterium* mediated infection. As the analysis was from transiently infiltrated leaves, a heterogeneous population of differentially methylated T-DNAs are likely to exist. Thus to ensure sufficient read depth as well as reduce the costs of analyzing multiple samples, NGS was employed and first explored using the Ion Torrent PGM.

Sequencing bisulfite amplicons from the 35S-eGFP+hp time course on the Ion Torrent PGM showed an increase in methylation over time (Figure 3A). However, at earlier time points of 2 and 3 dpi a large proportion of reads were truncated (Supplementary Table S3), most likely due to the read terminating when it approached a homopolymer track (Quail et al., 2012). This was confirmed when the fragmented reads were assembled against the reference. In the assembly, many of these reads terminated at homopolymer regions now created by bisulfite conversion of unmethylated DNA. This was especially noticeable at the region of the TATA box (data not presented). Similarly, termination of reads has been observed by Quail et al. (2012) when sequencing a highly AT-rich genome using the Ion Torrent platform.

To compare across the entire length of the amplicon, full length sequences generated by the Ion Torrent were selected by excluding all reads below 395 bp and with Phred scores equal to or below Q22. This inevitably biased reads, favoring a methylated state (lower homopolymers) especially during the earlier time points as the majority of reads were truncated. This bias is noticeable with the number of reads correlating to the level of methylation. For example, at 2 dpi, after filtering and employing a lowered Phred score of Q12 only 1143 out of 290,883 reads were available for analysis (0.39%) compared to 6734 out of 457,552 reads after filtering at 14 dpi with a higher Phred score of Q22 (1.47%). The methylation profile of this later time point at 14 dpi was then compared using a lower Phred score of Q12. Lowering this parameter resulted in 20,010 sequences (4.37% of available reads). This increase in the total number of reads did not result in a different observed methylation profile with 31% (CG), 64% (CHG), and 70% (CHH) cytosine methylation with Q22 compared to 32% (CG), and unchanged CHG and CHH cytosine methylation with Q12 (Figure 2). However, increasing the number of available reads by lowering the Phred score at earlier time points resulted in a lower methylation profile with sequences having a higher proportion of unmethylated cytosines (data not presented). As such, the Ion Torrent PGM is not suitable for highly unmethylated samples due to its bias of read termination at homopolymers. In addition, we found that less than 5% of all reads were available for methylation analysis after length and quality filtering (Table 1).

To utilize a higher proportion of the raw reads the bisulfite amplicons were sequenced on the Illumina MiSeq. Here two approaches were undertaken differing in the procedure used for adapter and index addition. Sequence reads originating from the MiSeq had a mean Phred score of approximately Q30 and this was used as a selection criterion. The first approach, tagmentation, randomly fragmented the bisulfite amplicons and then incorporated the Illumina Nextera XT v2 indices. As a result, the full length PCR product was only sequenced if tagmentation occurred at the product ends. Using this approach, raw reads totalled 374,865 sequences from the 14 dpi+hp sample, which were reduced to 80,940 (22% of available reads) after filtering with a minimum length of 250 bp out of the 418 bp (60%) and a Phred score of Q30 (Table 1).

This tagmentation approach was compared to the Ion Torrent sequencing platform for all time points in the 35S-eGFP+hp time course (Figure 3). The observed methylation profile trend is similar for both platforms showing an increase in methylation over the time course. Based on the MiSeq tagmentation results, there was no correlation between the number of reads and level of methylation as seen with the Ion Torrent. This indicates that sequencing on the MiSeq, unlike the Ion Torrent, did not cause the sequence to prematurely terminate at earlier time points due to homopolymers.

There is however a reduced methylation profile in the earlier time points (2–5 dpi) when analyzed using the Illumina platform. This may be because the highly unmethylated (AT-rich) sequences, previously excluded due to sequence fragmentation and quality score filtering, are now available for analysis. This difference is not seen at the later time points (7–21 dpi) most likely due to a higher proportion of highly methylated T-DNAs present in the amplicon. The benefit of using Tagmentation is that degenerate primers, already validated via Sanger sequencing, may be utilized when full length sequences are not needed. Stably integrated transgenes where the methylation marks are fixed will be suitable for such a sequencing approach.

To attain full length reads, a fusion approach involving adapters included on the degenerate primers as overhangs was used. The unique indexes were then added to the adapter sequences in a second round of PCR. Using this approach, the raw reads from the 14 dpi+hp sample totalled 19,942 sequences. After filtering for full length reads (395 bp) and a Phred score of Q30 there were 15,027 sequences (75%) available for methylation analysis (Table 1). This result showed the greatest improvement over both the Ion Torrent and tagmentation approach. At the 14 dpi+hp time point a reduced methylation profile of 31% (CG), 58% (CHG), and 64% (CHH) was observed in the tagmentation protocol when compared to the fusion protocol [31% (CG) 62% (CHG) and 70% (CHH) cytosine methylation]. This reduction was only evident in the CHG and CHH contexts. These differences could be due to the selection criteria of shorter 250 bp length reads compared to 395 bp.

### Extension Temperature

We determined that the intensity of T-DNA methylation in transiently infiltrated *N. benthamiana* leaves is dependent on the PCR extension temperature. Lowering the extension temperature between 65 and 68°C rather than the traditional 72°C for *Taq* polymerase results in efficient amplification of AT-rich samples (Yang et al., 2015). As bisulfite converted DNA is similar to AT-rich samples, when we lowered the extension temperature we found PCR bands of a higher intensity similar to that observed by Yang et al. (2015) (Figure 4B). In addition, the presence of primer-dimers was also reduced and this was more prominent from samples collected at the earlier time point of 2 dpi (Supplementary Figure S8). This indicates that more, possibly unmethylated T-DNA templates (AT-rich) were being efficiently amplified in the bisulfite converted DNA. A similar methylation profile was seen by both the suppliers (KAPA and NEB) at 67.6°C indicating that the extension temperature and not the PCR components affect the deduced methylation profile. In addition, the EpiMark Hot Start *Taq* polymerase (NEB), specifically formulated to amplify bisulfite converted templates has the recommended extension temperature of 68°C and as such was not tested at 72°C. These results indicate that there is a PCR bias toward methylated DNA sequences when extension takes place at 72°C when a range of differentially methylated T-DNA sequences exist within the DNA isolated from the infiltrated leaf patch. It is therefore likely that there may be fewer methylated T-DNAs in the transiently infiltrated leaf patch than the amplification profile generated from a 72°C PCR extension would suggest. To get a true sense of plant DNA methylation with reduced bias, methylation analysis of the sample should be performed without bisulfite conversion or PCR. With the improvement in long read NGS sequencing algorithms such as those employed by the Oxford Nanopore and Pacific Biosciences PacBio, such methylation analysis of plant DNA can one day accurately take place (Simpson et al., 2017). Although these algorithms are currently in their infancy, and are therefore inaccurate in differentiating methylated and unmethylated targets from a heterogeneous population (Barros-Silva et al., 2018). Until such a time, we suggest that lowering the PCR extension temperature following bisulfite conversion be taken into consideration when whole genome bisulfite sequencing (WGBS) is employed. This would be particularly relevant when treatments targeting a change in global methylation are induced such that a range of differentially methylated targets exist.

### T-DNA Methylation Evident Over a Time Course Regardless of the Experiment Undertaken

Depending on the length of time with the *vir* gene inducer, acetosyringone, prior to co-cultivation, transcription from T-DNAs can occur within 24 h (Narasimhulu et al., 1996). During this time the T-DNA must be excised from the *Agrobacterium* Ti plasmid, be transported into the plant cell, enter the nucleus and become double-stranded T-DNA (dsT-DNA) prior to transcription at a detectable level (Narasimhulu et al., 1996). Typically, transcription from infiltrated T-DNAs is known to peak around 2–3 dpi (Goodin et al., 2008; Bally et al., 2018) and this was also evident in our 35S-eGFP time course experiment (Figure 1C). Thus, if dsT-DNA can act as the substrate for transcription it could also act as a substrate for T-DNA methylation. Methylation of the promoter is known to disrupt the binding sites of transcription factors and RNA polymerase II (Chan et al., 2005; Brenet et al., 2011) and can lead to TGS. To determine the extent and time taken for T-DNA methylation in transiently infiltrated *N. benthamiana* leaves a time course assessing T-DNA methylation was carried out. T-DNA methylation was detected at 2–3 dpi in all time course experiments [35S-eGFP with and without hpRNA (Figure 5) and AtEF1α-A4 with and without the 5′ UTR intron (Figure 7)]. After 2–3 dpi, regardless of the experiment, a steady rate of T-DNA methylation accumulated over the first week post-infiltration. In the 35S-eGFP time courses this steady rate of methylation accumulation appears to plateau after 14 dpi (Figure 5). This plateau at the later time points may be due to the age of the leaf where regular biological functions, such as continued *de novo* and maintenance methylation, are not as active. It is already known that older *N. benthamiana* leaves have reduced leaf weight and protein content (Robert et al., 2013), and that in *N. tabacum* there is decreased potential for protein synthesis in older leaves compared to younger leaves (Buyel and Fischer, 2012).

### Methylation Distribution

The methylation profiles in the transient time course experiments show that methylation in the CHH context is the most prominent, followed by CHG and then CG (Figure 2, 3). This profile is in agreement with induced gene silencing and detection taking place in the same generation and is a hallmark of RdDM (Fultz and Slotkin, 2017). However, in literature where methylation has been assessed in stably integrated T-DNAs or endogenous genes, the inverse profile is seen with CG methylation being the most prominent (He et al., 2009; Haque et al., 2010; Mishiba et al., 2010; Dalakouras et al., 2012; Dadami et al., 2013, 2014; Du et al., 2015). The prominence of CG methylation in stable integrations is most likely due to its symmetrical context allowing for methylation maintenance. Following DNA replication, the daughter strands in the CG and CHG context are hemimethylated allowing for it to act as a template for CG and CHG methylation to be maintained by MET1 and CMT3, respectively (Wendte and Pikaard, 2017). However, in the asymmetrical context (CHH) there is an absence of the hemimethylated template. Thus, maintenance is not carried out by methyltransferase genes (*MET1* and *CMT3*) in these contexts and possibly another signal or trigger of asymmetrical methylation maintenance is required (Chan et al., 2005). In transiently infiltrated leaves, it appears that *de novo* T-DNA methylation is favored in the asymmetrical contexts possibly due to the conformational changes to the DNA (Klimasauskas et al., 1994) resulting in steric hindrance when MET1 is attempting to induce *de novo* methylation on both DNA strands.

### Addition of hpRNA Targeting eGFP

A time course containing a hairpin RNA (hpRNA) construct targeting the eGFP transgene was included in the co-infiltration solution and compared against the eGFP transgene alone (Figure 5). The hpRNA construct was included as it was believed that it would lead to dsRNA production and accelerate the T-DNA methylation such that it could be detected in the early stages post-infiltration. These dsRNA are the substrate required by the DICER-LIKE (DCL) proteins to generate the siRNAs involved in RNA directed DNA methylation (RdDM). The addition of the hpRNA did increase *de novo* T-DNA methylation compared to the treatment without the hpRNA and demonstrated the sensitivity of the developed assay.

Over the first week post-infiltration, there was a reduction in eGFP fluorescence and relative transcript abundance (Figure 1) coupled with a higher methylation profile in the hpRNA containing time course (*P* < 0.01 Figure 5). This indicated that the dsRNA production led to siRNA generation and expedited both PTGS and RdDM. However, these effects were short lived and no significant differences were observed at the later time point of 14 dpi (*P* > 0.05, Figure 5). During these later time points siRNA production may have been saturated such that the dsRNA produced from the hpRNA construct was no longer having an effect. Alternatively, it may be that the hpRNA was no longer being transcribed due to itself being silenced.

### T-DNA Methylation From a Heterogeneous Population

In our study, we isolated total DNA from agro-infiltrated *N. benthamiana* leaf patches across a time course. Each sample will be a heterogeneous population of T-DNAs from within the infiltrated *Agrobacterium* as well as differentially methylated T-DNAs from the plant cells. The T-DNA molecules with zero to low levels of cytosine methylation are probably largely from within the *Agrobacterium* as a study on *A. tumefaciens* strain C58 in *Arabidopsis* galls (predominantly bacterial rather than plant cells) estimated the cytosine methylation of T-DNA (for oncogenes *IaaH*, *IaaM*, and *Ipt*) to be less than 1% (Gohlke et al., 2013). During the early stages following agro-infiltration (2 and 3 dpi) a higher proportion of individual T-DNAs possess zero methylation than those at later time points (5, 7, 14, and 21 dpi) (Figure 6). However, a consistent level of low methylation (0, 1, and 2 methylated cytosines) from 5 to 21 dpi (*P* > 0.05, Figure 6) is coupled with a transition from medium to highly methylated T-DNAs (Figure 6). Taken together with the results presented in Figure 5 and Supplementary Figures S3, S4, this indicates that individual T-DNAs that are methylated at the earlier time points continue to accumulate further methylation as time progresses rather than the apparent increase in methylation being due to loss of unmethylated T-DNA from the declining levels of *Agrobacterium* in the samples.

### Inclusion of a 5′ UTR Intron

In studies by Meng et al. (2003, 2006) the inclusion of the first untranslated exon and intron of the maize ubiquitin1 promoter improved transgene stability in a large proportion of transgenic barley. Two siblings from a homozygous T_3_ population were identical in copy number and integration site. However, one sibling was silenced while the other remained transcriptionally active. Upon investigation, the silenced line had an increased and different methylation pattern at the 3′ end of the promoter. Thus, in this case, intron inclusion may not provide complete methylation protection to the promoter region and screening for stable lines may still need to take place albeit at a much lower frequency and the transcriptionally active line shows that the positive effects imparted by the intron is maintained across generations. Transgenes with introns within the coding sequence (CDS) tended to more stable, delayed onset of local and systemic silencing and produced less siRNAs (Vermeersch et al., 2010; Dadami et al., 2013).

Introns that provide enhancement to the transgenes increase transcription and promote translation (Rose, 2004). In our study, we included a 5′ UTR intron in the transgene and hypothesized that it would limit the spread of methylation to the promoter (transitivity) originating from the CDS by acting as a physical spacer. The elongation factor 1-alpha (EF1α) family in *Arabidopsis* consists of four genes (*AtEF1α-A1* through to *AtEF1α-A4*) and all possess a 5′ UTR intron. The A1 and A3 promoters with the 5′ UTR introns are required for high levels of gene expression (Curie et al., 1991, 1993; Chung et al., 2006). However, the A4 promoter is largely uncharacterized to date and is the highest expressing member based on EST and cDNA information (TAIR). The inclusion of the A4 promoter and its 5′ UTR intron resulted in a significant reduction in methylation of the promoter at 2, 3, and 4 dpi (*P* < 0.05, Figure 8). A reduction was also observed at 7 dpi but was not significantly different (*P* > 0.05). The reduction in promoter methylation indicates that the 5′ UTR intron may be acting as a physical barrier to impede transitivity. Alternatively, intron splicing may compete with the DNA methyltransferases thereby reducing T-DNA methylation. When the pre-mRNA is bound in the spliceosome complex to undergo intron splicing it is not available as a template for RNA-DEPENDENT RNA POLYMERASE 6 (RDR6) involved in the PTGS pathway to produce siRNAs (Christie et al., 2011). Thus, it is also not available to produce the 24 nt siRNAs needed for RdDM. In addition, the reduction in siRNA production against the transgene due to intron splicing could also account for the increase in fluorescence and *eGFP* transcript (Figure 7).

## Conclusion

One of the limitations in producing GM crops is the variability in transformation efficiency and transgene expression. Several methods have been explored to overcome this limitation as briefly discussed in the introduction. However, gene silencing still remains an issue. Because of this, large numbers of plants need to be screened to find the elite lines with stable and consistent transgene expression. This is possible for multinational companies but may be limiting for smaller research groups working on recalcitrant crops with limited plant growth space, and modest programs and budgets (Butaye et al., 2005; James, 2013).

Here we focused on DNA methylation as one of the factors contributing to the variability in producing GM crops. We report that *Agrobacterium* delivered T-DNAs undergo *de novo* methylation in the early stages following transformations into the leaves of *N. benthamiana* and as such may be responsible for the difficulties in obtaining consistent expression after transformation. For detecting methylation at base-pair resolution, we recommend the use of NGS to attain suitable read depth as a heterogeneous population of differentially methylated T-DNAs are likely to exist in these leaves. This transient assay, using the Illumina MiSeq platform, shows that the rate of *de novo* methylation can vary depending on the infiltration treatments and on the PCR extension temperature following bisulfite treatment. Mitigating *de novo* methylation of the T-DNA prior to integration is hypothesized to improve transformation efficiency by avoiding TGS. Thus, using the developed assay, several treatments and transgenic constructs can be rapidly and cost-effectively tested to ascertain the ideal conditions resulting in reduced *de novo* methylation as a way to improve transformation efficiency and potentially CRISPR-Cas9 editing in plants.

## Author Contributions

JP and RH designed the experiments. JP performed all experiments and analyzed all data. RH, PW, and KD supervised the experiments. JP wrote the original draft. JP, RH, PW, and KD reviewed and edited the manuscript.

## Conflict of Interest Statement

The authors declare that the research was conducted in the absence of any commercial or financial relationships that could be construed as a potential conflict of interest.

## References

[B1] Amasino R. M. Powell A. L. T. Gordon M. P. (1984). Changes in T-DNA methylation and expression are associated with phenotypic variation and plant regeneration in a crown gall tumor line. *Mol. Gen. Genet.* 197 437–446. 10.1007/bf00329940 6084805

[B2] Bally J. Jung H. Mortimer C. Naim F. Philips J. G. Hellens R. (2018). The rise and rise of *Nicotiana benthamiana*: a plant for all reasons. *Annu. Rev. Phytopathol.* 56 405–426. 10.1146/annurev-phyto-080417-050141 30149789

[B3] Bally J. Nakasugi K. Jia F. Jung H. Ho S. Y. W. Wong M. (2015). The extremophile *Nicotiana benthamiana* has traded viral defence for early vigour. *Nat. Plants* 1:15165. 10.1038/nplants.2015.165 27251536

[B4] Barros-Silva D. Marques C. Henrique R. Jerónimo C. (2018). Profiling DNA methylation based on next-generation sequencing approaches: new insights and clinical applications. *Genes* 9:429. 10.3390/genes9090429 30142958 PMC6162482

[B5] Bolger A. M. Lohse M. Usadel B. (2014). Trimmomatic: a flexible trimmer for illumina sequence data. *Bioinformatics* 30 2114–2120. 10.1093/bioinformatics/btu170 24695404 PMC4103590

[B6] Brenet F. Moh M. Funk P. Feierstein E. Viale A. J. Socci N. D. (2011). DNA methylation of the first exon is tightly linked to transcriptional silencing. *PLoS One* 6:e14524. 10.1371/journal.pone.0014524 21267076 PMC3022582

[B7] Brouwer C. Bruce W. Maddock S. Avramova Z. Bowen B. (2002). Suppression of transgene silencing by matrix attachment regions in maize: a dual role for the maize 5’ ADH1 matrix attachment region. *Plant Cell* 14:2251. 10.1105/tpc.004028 12215518 PMC150768

[B8] Bustin S. A. Benes V. Garson J. A. Hellemans J. Huggett J. Kubista M. (2009). The MIQE guidelines: minimum information for publication of quantitative real-time PCR experiments. *Clin. Chem.* 55 611–622. 10.1373/clinchem.2008.112797 19246619

[B9] Butaye K. M. J. Cammue B. P. A. Delauré S. L. De Bolle M. F. C. (2005). Approaches to minimize variation of transgene expression in plants. *Mol. Breed.* 16 79–91. 10.1007/s11032-005-4929-9

[B10] Buyel J. F. Fischer R. (2012). Predictive models for transient protein expression in tobacco (*Nicotiana tabacum L.*) can optimize process time, yield, and downstream costs. *Biotechnol. Bioeng.* 109 2575–2588. 10.1002/bit.24523 22511291

[B11] Cao X. Aufsatz W. Zilberman D. Mette M. F. Huang M. S. Matzke M. (2003). Role of the *DRM* and *CMT3* methyltransferases in RNA-directed DNA methylation. *Curr. Biol.* 13 2212–2217. 10.1016/j.cub.2003.11.052 14680640

[B12] Cao X. Jacobsen S. E. (2002). Role of the *Arabidopsis DRM* methyltransferases in de novo DNA methylation and gene silencing. *Curr. Biol.* 12 1138–1144. 10.1016/S0960-9822(02)00925-9 12121623

[B13] Cervera M. Pina J. A. Juárez J. Navarro L. Peña L. (1998). Agrobacterium-mediated transformation of citrange: factors affecting transformation and regeneration. *Plant Cell Rep.* 18 271–278. 10.1007/s002990050570 30744234

[B14] Chan S. W. L. Henderson I. R. Jacobsen S. E. (2005). Gardening the genome: DNA methylation in *Arabidopsis thaliana*. *Nat. Rev. Genet.* 6 351–360. 10.1038/nrg1601 15861207

[B15] Christie M. Croft L. J. Carroll B. J. (2011). Intron splicing suppresses RNA silencing in *Arabidopsis*. *Plant J.* 68 159–167. 10.1111/j.1365-313X.2011.04676.x 21689169

[B16] Chung B. Simons C. Firth A. Brown C. Hellens R. (2006). Effect of 5’UTR introns on gene expression in *Arabidopsis thaliana*. *BMC Genomics* 7:120. 10.1186/1471-2164-7-120 16712733 PMC1482700

[B17] Clough S. J. Bent A. F. (1998). Floral dip: a simplified method for *Agrobacterium*-mediated transformation of *Arabidopsis thaliana*. *Plant J.* 16 735–743. 10.1046/j.1365-313x.1998.00343.x 10069079

[B18] Coutu C. Brandle J. Brown D. Brown K. Miki B. Simmonds J. (2007). pORE: a modular binary vector series suited for both monocot and dicot plant transformation. *Transgenic Res.* 16 771–781. 10.1007/s11248-007-9066-2 17273915

[B19] Curie C. Axelos M. Bardet C. Atanassova R. Chaubet N. Lescure B. (1993). Modular organization and developmental activity of an *Arabidopsis thaliana* EF-1α gene promoter. *Mol. Gen. Genet.* 238 428–436. 10.1007/bf00292002 8492811

[B20] Curie C. Liboz T. Bardet C. Gander E. Médale C. Axelos M. (1991). *Cis* and trans-acting elements involved in the activation of *Arabidopsis thaliana* A1 gene encoding the translation elongation factor EF-1a. *Nucleic Acids Res.* 19 1305–1310. 10.1093/nar/19.6.1305 1840652 PMC333858

[B21] Dadami E. Dalakouras A. Zwiebel M. Krczal G. Wassenegger M. (2014). An endogene-resembling transgene is resistant to DNA methylation and systemic silencing. *RNA Biol.* 11 934–941. 10.4161/rna.29623 25180820 PMC4179966

[B22] Dadami E. Moser M. Zwiebel M. Krczal G. Wassenegger M. Dalakouras A. (2013). An endogene-resembling transgene delays the onset of silencing and limits siRNA accumulation. *FEBS Lett.* 587 706–710. 10.1016/j.febslet.2013.01.045 23380068

[B23] Dalakouras A. Dadami E. Zwiebel M. Krczal G. Wassenegger M. (2012). Transgenerational maintenance of transgene body CG but not CHG and CHH methylation. *Epigenetics* 7 1071–1078. 10.4161/epi.21644 22863736 PMC3466191

[B24] Du J.-L. Zhang S.-W. Huang H.-W. Cai T. Li L. Chen S. (2015). The splicing factor PRP31 is involved in transcriptional gene silencing and stress response in *Arabidopsis*. *Mol. Plant* 8 1053–1068. 10.1016/j.molp.2015.02.003 25684655

[B25] Forsbach A. Schubert D. Lechtenberg B. Gils M. Schmidt R. (2003). A comprehensive characterization of single-copy T-DNA insertions in the *Arabidopsis thaliana* genome. *Plant Mol. Biol.* 52 161–176. 10.1023/a:1023929630687 12825697

[B26] Fultz D. Slotkin R. K. (2017). Exogenous transposable elements circumvent identity-based silencing, permitting the dissection of expression-dependent silencing. *Plant Cell* 29 360–376. 10.1105/tpc.16.00718 28193737 PMC5354191

[B27] Gelvin S. B. Karcher S. J. DiRita V. J. (1983). Methylation of the T-DNA in *Agrobacterium tumefaciens* and in several crown gall tumors. *Nucleic Acids Res.* 11 159–174. 10.1093/nar/11.1.159 6306562 PMC325696

[B28] Gohlke J. Scholz C.-J. Kneitz S. Weber D. Fuchs J. Hedrich R. (2013). DNA methylation mediated control of gene expression is critical for development of crown gall tumors. *PLoS Genetics* 9:e1003267. 10.1371/journal.pgen.1003267 23408907 PMC3567176

[B29] Goodin M. M. Zaitlin D. Naidu R. A. Lommel S. A. (2008). *Nicotiana benthamiana*: its history and future as a model for plant–pathogen interactions. *Mol. Plant Microbe Interact.* 21 1015–1026. 10.1094/MPMI-21-8-1015 18616398

[B30] Gruntman E. Qi Y. Slotkin R. K. Roeder T. Martienssen R. A. Sachidanandam R. (2008). Kismeth: analyzer of plant methylation states through bisulfite sequencing. *BMC Bioinformatics* 9:371. 10.1186/1471-2105-9-371 18786255 PMC2553349

[B31] Haque N. Yamaoka N. Nishiguchi M. (2010). Spreading of RNA silencing and DNA methylation in transgenic hybrid plants with the coat protein gene of Sweet potato feathery mottle virus. *Breed. Sci.* 60 361–370. 10.1270/jsbbs.60.361

[B32] He X.-J. Hsu Y.-F. Zhu S. Wierzbicki A. T. Pontes O. Pikaard C. S. (2009). An effector of RNA-directed DNA methylation in *Arabidopsis* is an ARGONAUTE 4- and RNA-binding protein. *Cell* 137 498–508. 10.1016/j.cell.2009.04.028 19410546 PMC2700824

[B33] Helliwell C. Waterhouse P. (2003). Constructs and methods for high-throughput gene silencing in plants. *Methods* 30 289–295. 10.1016/S1046-2023(03)00036-7 12828942

[B34] James C. (2013). Global status of commercialized biotech/GM crops: 2013. *ISAAA Brief No.* 46 ISAAA: Ithaca, NY.

[B35] James D. J. Uratsu S. Cheng J. Negri P. Viss P. Dandekar A. M. (1993). Acetosyringone and osmoprotectants like betaine or proline synergistically enhance *Agrobacterium*-mediated transformation of apple. *Plant Cell Rep.* 12 559–563. 10.1007/bf00233060 24201785

[B36] Jin S. Zhang X. Liang S. Nie Y. Guo X. Huang C. (2005). Factors affecting transformation efficiency of embryogenic callus of Upland cotton (*Gossypium hirsutum*) with *Agrobacterium tumefaciens*. *Plant Cell Tissue Organ Cult.* 81 229–237. 10.1007/s11240-004-5209-9

[B37] Kim S.-I. Gelvin S. B. (2007). Genome-wide analysis of *Agrobacterium* T-DNA integration sites in the *Arabidopsis* genome generated under non-selective conditions. *Plant J.* 51 779–791. 10.1111/j.1365-313X.2007.03183.x 17605756

[B38] Klimasauskas S. Kumar S. Roberts R. J. Cheng X. (1994). Hhal methyltransferase flips its target base out of the DNA helix. *Cell* 76 357–369. 10.1016/0092-8674(94)90342-5 8293469

[B39] Law J. A. Jacobsen S. E. (2010). Establishing, maintaining and modifying DNA methylation patterns in plants and animals. *Nat. Rev. Genet.* 11 204–220. 10.1038/nrg2719 20142834 PMC3034103

[B40] Laxa M. (2017). Intron-mediated enhancement: a tool for heterologous gene expression in plants. *Front. Plant Sci.* 7:1977. 10.3389/fpls.2016.01977 28111580 PMC5216049

[B41] Laxa M. Müller K. Lange N. Doering L. Pruscha J. T. Peterhänsel C. (2016). The 5’UTR intron of *Arabidopsis* GGT1 aminotransferase enhances promoter activity by recruiting RNA polymerase II. *Plant Physiol.* 172:313. 10.1104/pp.16.00881 27418588 PMC5074633

[B42] Li J. Brunner A. M. Meilan R. Strauss S. H. (2008). Matrix attachment region elements have small and variable effects on transgene expression and stability in field-grown Populus. *Plant Biotechnol. J.* 6 887–896. 10.1111/j.1467-7652.2008.00369.x 19548343

[B43] Liu D. Shi L. Han C. Yu J. Li D. Zhang Y. (2012). Validation of reference genes for gene expression studies in virus-infected *Nicotiana benthamiana* using quantitative real-time PCR. *PLoS One* 7:e46451. 10.1371/journal.pone.0046451 23029521 PMC3460881

[B44] Lizamore D. Winefield C. (2014). The addition of an organosilicone surfactant to *Agrobacterium* suspensions enables efficient transient transformation of in vitro grapevine leaf tissue at ambient pressure. *Plant Cell Tissue Organ Cult.* 120 607–615. 10.1007/s11240-014-0627-9

[B45] Martin M. (2011). Cutadapt removes adapter sequences from high-throughput sequencing reads. *EMBnet J.* 17 10–12. 10.14806/ej.17.1.200

[B46] Mascarenhas D. Mettler I. J. Pierce D. A. Lowe H. W. (1990). Intron-mediated enhancement of heterologous gene expression in maize. *Plant Mol. Biol.* 15 913–920. 10.1007/bf00039430 2103480

[B47] Matzke M. A. Mosher R. A. (2014). RNA-directed DNA methylation: an epigenetic pathway of increasing complexity. *Nat. Rev. Genet.* 15 394–408. 10.1038/nrg3683 24805120

[B48] McHale M. Eamens A. L. Finnegan E. J. Waterhouse P. M. (2013). A 22-nt artificial microRNA mediates widespread RNA silencing in *Arabidopsis*. *Plant J.* 76 519–529. 10.1111/tpj.12306 23937661 PMC4241025

[B49] Meng L. Bregitzer P. Zhang S. Lemaux P. (2003). Methylation of the exon/intron region in the Ubi1 promoter complex correlates with transgene silencing in barley. *Plant Mol. Biol.* 53 327–340. 10.1023/B:PLAN.0000006942.00464.e3 14750522

[B50] Meng L. Ziv M. Lemaux P. (2006). Nature of stress and transgene locus influences transgene expression stability in barley. *Plant Mol. Biol.* 62 15–28. 10.1007/s11103-006-9000-7 16900326

[B51] Mishiba K. I. Yamasaki S. Nakatsuka T. Abe Y. Daimon H. Oda M. (2010). Strict de novo methylation of the 35S enhancer sequence in gentian. *PLoS One* 5:e9670. 10.1371/journal.pone.0009670 20351783 PMC2843634

[B52] Morello L. Breviario D. (2008). Plant spliceosomal introns: not only cut and paste. *Curr. Genom.* 9 227–238. 10.2174/138920208784533629 19452040 PMC2682935

[B53] Narasimhulu S. B. Deng X. B. Sarria R. Gelvin S. B. (1996). Early transcription of *Agrobacterium* T-DNA genes in tobacco and maize. *Plant Cell* 8 873–886. 10.2307/3870289 8672885 PMC161145

[B54] Nocarova E. Opatrny Z. Fischer L. (2010). Successive silencing of tandem reporter genes in potato (*Solanum tuberosum*) over 5 years of vegetative propagation. *Ann. Bot.* 106 565–572. 10.1093/aob/mcq153 20829194 PMC2944976

[B55] Philips J. G. Naim F. Lorenc M. T. Dudley K. J. Hellens R. P. Waterhouse P. M. (2017). The widely used *Nicotiana benthamiana* 16c line has an unusual T-DNA integration pattern including a transposon sequence. *PLoS One* 12:e0171311. 10.1371/journal.pone.0171311 28231340 PMC5322946

[B56] Quail M. A. Smith M. Coupland P. Otto T. D. Harris S. R. Connor T. R. (2012). A tale of three next generation sequencing platforms: comparison of Ion Torrent, Pacific Biosciences and Illumina MiSeq sequencers. *BMC Genomics* 13:341. 10.1186/1471-2164-13-341 22827831 PMC3431227

[B57] Robert S. Khalf M. Goulet M.-C. D’Aoust M.-A. Sainsbury F. Michaud D. (2013). Protection of recombinant mammalian antibodies from development-dependent proteolysis in leaves of *Nicotiana benthamiana*. *PLoS One* 8:e70203. 10.1371/journal.pone.0070203 23894618 PMC3720903

[B58] Rose A. B. (2004). The effect of intron location on intron-mediated enhancement of gene expression in *Arabidopsis*. *Plant J.* 40 744–751. 10.1111/j.1365-313X.2004.02247.x 15546357

[B59] Rose A. B. Elfersi T. Parra G. Korf I. (2008). Promoter-proximal introns in *Arabidopsis thaliana* are enriched in dispersed signals that elevate gene expression. *Plant Cell* 20:543. 10.1105/tpc.107.057190 18319396 PMC2329928

[B60] Saze H. Scheid O. M. Paszkowski J. (2003). Maintenance of CpG methylation is essential for epigenetic inheritance during plant gametogenesis. *Nat. Genet.* 34:65. 10.1038/ng1138 12669067

[B61] Sharma M. K. Solanke A. U. Jani D. Singh Y. Sharma A. K. (2009). A simple and efficient *Agrobacterium*-mediated procedure for transformation of tomato. *J. Biosci.* 34:423 10.1007/s12038-009-0049-8 19805904

[B62] Shrawat A. K. Lörz H. (2006). *Agrobacterium*-mediated transformation of cereals: a promising approach crossing barriers. *Plant Biotechnol. J.* 4 575–603. 10.1111/j.1467-7652.2006.00209.x 17309731

[B63] Sijen T. Vijn I. Rebocho A. van Blokland R. Roelofs D. Mol J. N. M. (2001). Transcriptional and posttranscriptional gene silencing are mechanistically related. *Curr. Biol.* 11 436–440. 10.1016/S0960-9822(01)00116-6 11301254

[B64] Simpson J. T. Workman R. E. Zuzarte P. C. David M. Dursi L. J. Timp W. (2017). Detecting DNA cytosine methylation using nanopore sequencing. *Nat. Methods* 14:407. 10.1038/nmeth.4184 28218898

[B65] Singer K. Shiboleth Y. M. Li J. Tzfira T. (2012). Formation of complex extrachromosomal T-DNA structures in *Agrobacterium tumefaciens*-infected plants. *Plant Physiol.* 160 511–522. 10.1104/pp.112.200212 22797657 PMC3440224

[B66] Singer S. D. Liu Z. Cox K. D. (2012). Minimizing the unpredictability of transgene expression in plants: the role of genetic insulators. *Plant Cell Rep.* 31 13–25. 10.1007/s00299-011-1167-y 21987122

[B67] Tashiro R. M. Philips J. G. Winefield C. S. (2016). Identification of suitable grapevine reference genes for qRT-PCR derived from heterologous species. *Mol. Genet. Genom.* 291 483–492. 10.1007/s00438-015-1081-z 26129768

[B68] van Leeuwen W. Hagendoorn M. J. M. Ruttink T. van Poecke R. van Der Plas L. H. W. van Der Krol A. R. (2000). The use of the luciferase reporter system forin planta gene expression studies. *Plant Mol. Biol. Rep.* 18 143–144. 10.1007/bf02824024

[B69] van Leeuwen W. Mlynárová L. Nap J. P. van der Plas L. H. W. van der Krol A. R. (2001). The effect of MAR elements on variation in spatial and temporal regulation of transgene expression. *Plant Mol. Biol.* 47 543–554. 10.1023/a:1011840310436 11669579

[B70] Vermeersch L. De Winne N. Depicker A. (2010). Introns reduce transitivity proportionally to their length, suggesting that silencing spreads along the pre-mRNA. *Plant J.* 64 392–401. 10.1111/j.1365-313X.2010.04335.x 21049564

[B71] Voinnet O. Rivas S. Mestre P. Baulcombe D. (2003). Retracted: an enhanced transient expression system in plants based on suppression of gene silencing by the p19 protein of tomato bushy stunt virus. *Plant J.* 33 949–956. 10.1046/j.1365-313X.2003.01676.x 12609035

[B72] Wendte J. M. Pikaard C. S. (2017). The RNAs of RNA-directed DNA methylation. *Biochim. Biophys. Acta* 140–148. 10.1016/j.bbagrm.2016.08.004 27521981 PMC5203809

[B73] Wise A. A. Liu Z. Binns A. N. (2006). “Three methods for the introduction of foreign DNA into *Agrobacterium*,” in *Agrobacterium Protocols*, ed. Wang K. (Totowa, NJ: Humana Press), 43–54. 10.1385/1-59745-130-4:43 16988332

[B74] Wydro M. Kozubek E. Lehmann P. (2006). Optimization of transient *Agrobacterium*-mediated gene expression system in leaves of *Nicotiana benthamiana*. *Acta Biochim. Pol.* 53 289–298. 16582986

[B75] Yang Y. Li R. Qi M. (2000). In vivo analysis of plant promoters and transcription factors by agroinfiltration of tobacco leaves. *Plant J.* 22 543–551. 10.1046/j.1365-313x.2000.00760.x 10886774

[B76] Yang Y. Sebra R. Pullman B. S. Qiao W. Peter I. Desnick R. J. (2015). Quantitative and multiplexed DNA methylation analysis using long-read single-molecule real-time bisulfite sequencing (SMRT-BS). *BMC Genomics* 16:350. 10.1186/s12864-015-1572-7 25943404 PMC4422326

[B77] Zhang J. Kobert K. Flouri T. Stamatakis A. (2014). PEAR: a fast and accurate Illumina Paired-End reAd mergeR. *Bioinformatics* 30 614–620. 10.1093/bioinformatics/btt593 24142950 PMC3933873

