# Supplementary material for: The Rapid Methylation of T-DNAs Upon Agrobacterium Inoculation in Plant Leaves
Source: Front Plant Sci. 2019 Mar 15;10:312. doi: 10.3389/fpls.2019.00312 (PMC6428780; doi:10.3389/fpls.2019.00312)
Supplement: Supplementary file 1 [file Data_Sheet_1.PDF]

frontiers

Supplementary Material

The Rapid Methylation of T-DNAs upon *Agrobacterium* Inoculation in Plant Leaves

Joshua G. Philips*, Kevin J. Dudley, Peter M. Waterhouse, Roger P. Hellens

*** Correspondence:** Joshua G. Philips: j.philips@qut.edu.au

1 Supplementary Tables and Figures

1.1 Supplementary Tables

Supplementary Table S1. Primers and Ion Torrent barcode sequences used**.**

Supplementary Table S2. Number of filtered reads from three biologically independent pooled leaf infiltrations that were used to generate the cytosine methylation profile presented in Figure 5 and Figure 7.

Supplementary Table S3. Reads attained by sequencing the 35S-eGFP+hp time course using the Ion Torrent PGM. Reads above 395 bp prior to Phred score filtering were used for analysis. The blue line shows the 395 bp marker on the read length graph with anything to left of the line appearing as truncated reads.

1.2 Supplementary Figures

Supplementary Figure S1. DNA sequence containing the 35S promoter, eGFP coding sequence and OCS transcriptional terminator introduced into the pORE 03 backbone (Coutu et al., 2007) via the HindIII and EcoRI restriction sites.

Supplementary Figure S2. (Pages 7-12) Images of *N. benthamiana* leaves infiltrated with 35S-eGFP (top panel) and infiltrated with 35S-eGFP+hp (bottom panel). **(Pages 13-17)** Images of *N. benthamiana* leaves infiltrated with AtEF1α-A4+Intron-eGFP (top panel) and infiltrated with AtEF1α-A4–Intron-eGFP (bottom panel). Fluorescent images were captured by applying the Dark Reader Hand Lamp HL32T (Clare Chemical) using the Canon EOS 550D DLSR camera affixed with an EF-S 60 mm lens and a Hoya HMC O(G) amber filter blocking blue light transmission. Images were then converted to black-and-white and subjected to ImageJ analysis as described.

Supplementary Figure S3. Cytosine context specific methylation analysis comparison in the time course of the 35S-eGFP transgene with and without a hairpin. Comparisons carried out by ANOVA. Statistically significant differences, $*P < 0.05$, $**P < 0.01$. n = 3 biological replicates, means ± SEM. The black bar in the gene cartoon represents the 418 bp region analysed by bisulfite PCR.

Supplementary Figure S4. Percentage cytosine methylation in the T-DNA of the 35S-eGFP transgene with and without the hpRNA. The black bar represents a 418 bp region analysed by bisulfite PCR. The blue region represents the 35S promoter, green region is the eGFP coding region, purple region is region targeting eGFP by hpRNA and the black region is the degenerate cytosine sites of the primers.

Supplementary Figure S5. (A) RT-PCR using primers EF1a TSS F→dBS R7.1 to test intron processing. Expected sizes: processed 176 bp, unprocessed 675 bp. Diluted minipreparation of the pEF1a-4 +Intron plasmid was used as a positive control for the unprocessed state. 100 bp DNA Ladder (GeneRuler). **(B and C)** RT-PCR using primers NbL23qPCR F→NbL23 qPCR R to test for residual gDNA contamination after DNaseI treatment of RNA. Expected sizes: cDNA 110 bp, gDNA 338 bp. Hyperladder 25bp (Bioloine)

Supplementary Figure S6. Cytosine context specific methylation analysis comparison in the time course of the *AtEF1α-A4* promoter regulating eGFP with and without the 5'UTR intron. Comparisons carried out by ANOVA. Statistically significant differences, $*P < 0.05$, $***P < 0.001$. n = 3 biological replicates, means ± SEM. The black bar in the gene cartoon represents the 546 bp region analysed by bisulfite PCR.

Supplementary Figure S7. Percentage cytosine methylation in the T-DNA of the AtEF1α-eGFP transgene with and without the intron. The black bar represents a 546 bp region analysed by bisulfite PCR. The blue region represents the AtEF1α promoter and the black region is the degenerate cytosine sites of the primers.

Supplementary Figure S8. Gel electrophoresis showing amplification intensity when differing PCR extension temperatures are used with bisulfite DNA as the template. **(A)** Amplification using dBS F4.1 and dBS R7.1 primers with pooled bisulfite converted DNA (2-21 dpi+hp) as template **(B)** Amplification using dBS F4.1 and dBS R7.1 primers with bisulfite converted DNA from the 2 dpi+hp time point as template. **(C)** Amplification using dBS F4.1 Illumina and dBS R7.1 Illumina primers from the 14 dpi+hp time point as template, also presented in Figure 4 B. Arrows indicate primer-dimers. 100 bp DNA Ladder (GeneRuler).

frontiers

Supplementary Table S1. Primers and Ion Torrent barcode sequences used.

**Primer Name**	**Sequence**	**Description**	**Amplicon**
dBS F4.1	AGYTATYTGTYAYTTYATYGAAAGGAYAGTAG	Degenerate primer for 35S-eGFP from Mishiba et al., (2010) modified	418 bp
dBS R7.1	ATCRCCCTCRCCCTCRCCRRACAC	Degenerate primer for 35S-eGFP from Mishiba et al., (2010)	
dBS F4.1 Adtp A Bar 1-20	CCATCTCATCCCTGCGTGTCTCCGACTCAGXXXXXXXXXXTNNNNNNAGYTATYTGTYAYTTYATYGAAAGGAYAGTAG	dBS F4.1 Adapter A Barcodes 1-20 for Ion Torrent, where barcoded sequence is replaced by XXXXXXXXXX	418 bp
dBS R7.1 Adapter trP1	CCTCTCTATGGGCAGTCGGTGATATCRCCCTCRCCCTCRCCRRACAC	dBS R7.1 Adapter trP1 for Ion Torrent	
dBS F4.1 Illumina	TCGTCGGCAGCGTCAGATGTGTATAAGAGACAGAGYTATYTGTYAYTTYATYGAAAGGAYAGTAG	dBS F4.1 for Illumina Nextera XT	418 bp
dBS R7.1 Illumina	GTCTCGTGGGCTCGGAGATGTGTATAAGAGACAGATCRCCCTCRCCCTCRCCRRACAC	dBS R7.1 for Illumina Nextera XT	
EF1a-4 Prom GA F	CGGGCGCCCCGCGGAAAGCTTATTTGCGAGTATAATCTTTGAACT	Cloning F of AtEf1a-4 Promoter with Gibson into p35S-eGFP	1457 bp
EF1a-4 Prom +Int GA R	CTCACCATTGAATTCCTCGAAGCTGTCAAAACAAAAACAAAA	Cloning R of AtEf1a-4 Promoter -Int Gibson into p35S-eGFP	
EF1a-4 Prom -Int+CT GA R	CTCACCATTGAATTCCTCGAAGCTGCAAGTAAGAATCTGAAAG	Cloning R of AtEf1a-4 Promoter -Int+CT with Gibson into p35S-eGFP	958 bp
EF1a-4 TSS F	ATTTCTCACATTTTCGTAGCCG	Forward primer to test for intron splicing	176 bp cDNA
dBS R7.1	ATCRCCCTCRCCCTCRCCRRACAC	Reverse primer to test for intron splicing	675 bp gDNA
GFP qPCR F	CGACCACTACCAGCAGAACA	qPCR primer eGFP F 2step 60°C	133 bp
GFP qPCR R	GAACTCCAGCAGGACCATGT	qPCR primer eGFP R 2step 60°C	
NbL23 qPCR F	AAGGATGCCGTGAAGAAGATGT	qPCR primer from Liu et al., 2012	110 bp cDNA
NbL23 qPCR R	GCATCGTAGTCAGGAGTCAACC	qPCR primer from Liu et al., 2012	338 bp gDNA
NbPP2A qPCR F	GACCCTGATGTTGATGTTCGCT	qPCR primer from Liu et al., 2012	123 bp
NbPP2A qPCR R	GAGGGATTTGAAGAGAGATTTC	qPCR primer from Liu et al., 2012	

**Barcode**	**Sequence**	**Barcode**	**Sequence**
Barcode 1	CTAAGGTAAC	Barcode 11	TCCTCGAATC
Barcode 2	TAAGGAGAAC	Barcode 12	TAGGTGGTTC
Barcode 3	AAGAGGATTC	Barcode 13	TCTAACGGAC
Barcode 4	TACCAAGATC	Barcode 14	TTGGAGTGTC
Barcode 5	CAGAAGGAAC	Barcode 15	TCTAGAGGTC
Barcode 6	CTGCAAGTTC	Barcode 16	TCTGGATGAC
Barcode 7	TTCGTGATTC	Barcode 17	TCTATTCGTC
Barcode 8	TTCCGATAAC	Barcode 18	AGGCAATTGC
Barcode 9	TGAGCGGAAC	Barcode 19	TTAGTCGGAC
Barcode 10	CTGACCGAAC	Barcode 20	CAGATCCATC

Note: Sequence in green is gene specific, amplicon sizes are listed without overhangs. Sequences in red for EF1a-4 primers are overhangs for Gibson assembly into the p35S-eGFP vector (Supplementary Figure S1)

References not shown in the main text

Mishiba, K.-i., Yamasaki, S., Nakatsuka, T., Abe, Y., Daimon, H., Oda, M., et al. (2010). Strict *de novo* methylation of the 35S enhancer sequence in gentian. *PLOS ONE* 5(3), e9670. doi: 10.1371/journal.pone.0009670.

Supplementary Table S2. Number of filtered reads from three biologically independent pooled leaf infiltrations that were used to generate the cytosine methylation profile presented in Figure 5 and Figure 7.

Sample	Reads	Sample	Reads	Sample	Reads	Sample	Reads
35S-eGFP-hp 2 dpi	20,778, 41,623, 32,676	35S-eGFP+hp 2 dpi	28,867, 19,640, 23,237	+ 5'UTR Intron 2 dpi	11,987, 6355, 11,377	- 5'UTR Intron 2 dpi	4765, 9088, 5258
35S-eGFP-hp 3 dpi	23,753, 24,958, 39,415	35S-eGFP+hp 3 dpi	38,268, 19,675, 35,840	+ 5'UTR Intron 3 dpi	9648, 10,792, 9574	- 5'UTR Intron 3 dpi	4707, 1180, 2431
35S-eGFP-hp 5 dpi	27,030, 13,084, 26,507	35S-eGFP+hp 5 dpi	23,350, 14,607, 22,658	+ 5'UTR Intron 4 dpi	8921, 13,300, 4701	- 5'UTR Intron 4 dpi	6860, 4585, 3786
35S-eGFP-hp 7 dpi	19,342, 18,927, 21,969	35S-eGFP+hp 7 dpi	26,736, 27,121, 18,257	+ 5'UTR Intron 5 dpi	5716, 5441, 5836	- 5'UTR Intron 5 dpi	3146, 8153, 6386
35S-eGFP-hp 14 dpi	26,230, 23,412, 8589	35S-eGFP+hp 14 dpi	21,732, 23,764, 20,069	+ 5'UTR Intron 7 dpi	8136, 6580, 2784	- 5'UTR Intron 7 dpi	2819, 3936, 8417
35S-eGFP-hp 21 dpi	31,301, 35,395, 27,550	35S-eGFP+hp 21 dpi	28,689, 37,780, 34,049				
35S-eGFP-hp 28 dpi	22,131, 32,600, 27,419	35S-eGFP+hp 28 dpi	31,453, 27,760, 34,800				

Supplementary Table S3. Reads attained by sequencing the 35S-eGFP+hp time course using the Ion Torrent PGM. Reads above 395 bp prior to Phred score filtering were used for analysis. The blue line shows the 395 bp marker on the read length graph with anything to left of the line appearing as truncated reads.

Time Point	Total Reads	Reads above 395 bp	Percentage	Read length graph
2 dpi	290,883	10,973	3.8%	
3 dpi	327,395	15,163	4.6%	
5 dpi	344,467	39,931	11.6%	
7 dpi	382,225	70,945	18.6%	
14 dpi	457,552	85,942	18.8%	
21 dpi	539,826	137,392	25.5%	

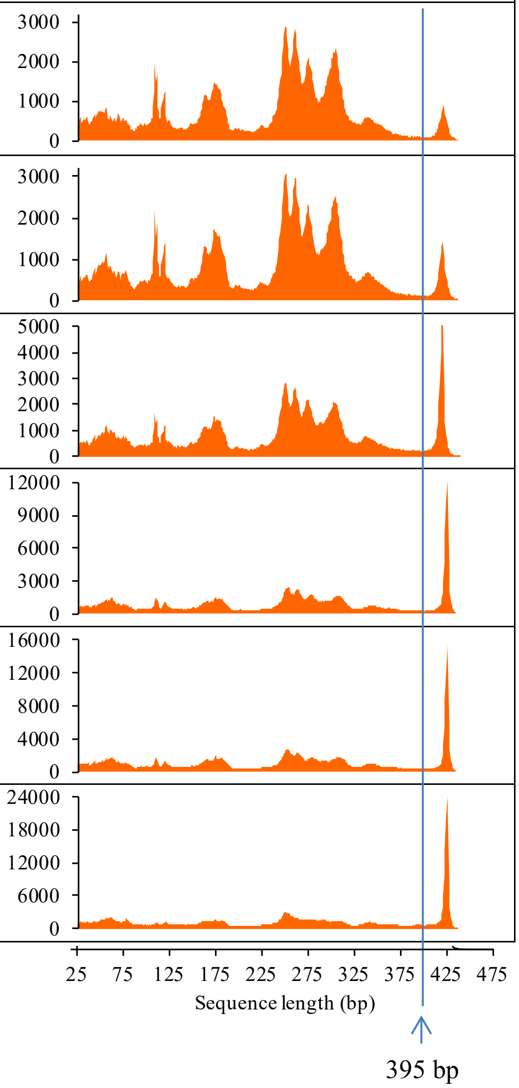

AAGCTTGCTCGACGAATTAATTCCAATCCCACAAAAATCTGAGCTTAACAGCACAGTTGCTCCTCTCAGAGCAGAATC
GGGTATTCAACACCCTCATATCAACTACTACGTTGTGTATAACGGTCCACATGCCGGTATATACGATGACTGGGGTTG
TACAAAGGCGGCAACAAACGGCGTTCCCGGAGTTGCACACAAGAAATTTGCCACTATTACAGAGGCAAGAGCAGCAGC
TGACGCGTACACAACAAGTCAGCAAACAGACAGGTTGAACTTCATCCCCAAAGGAGAAGCTCAACTCAAGCCCAAGAG
CTTTGCTAAGGCCCTAACAAGCCCACCAAAGCAAAAAGCCCACTGGCTCACGCTAGGAACCAAAAGGCCCAGCAGTGA
TCCAGCCCCAAAAGAGATCTCCTTTGCCCCGGAGATTACAATGGACGATTTCCTCTATCTTTACGATCTAGGAAGGAA
GTTCGAAGGTGAAGGTGACGACACTATGTTCACCACTGATAATGAGAAGGTTAGCCTCTTCAATTTCAGAAAGAATGC
TGACCCACAGATGGTTAGAGAGGCCTACGCAGCAGGTCTCATCAAGACGATCTACCCGAGTAACAATCTCCAGGAGAT
CAAATACCTTCCCAAGAAGGTTAAAGATGCAGTCAAAAGATTCAGGACTAATTGCATCAAGAACACAGAGAAAGACAT
ATTTCTCAAGATCAGAAGTACTATTCCAGTATGGACGATTCAAGGCTTGCTTCATAAACCAAGGCAAGTAATAGAGAT
TGGAGTCTCTAAAAAGGTAGTTCCTACTGAATCTAAGGCCATGCATGGAGTCTAAGATTCAAATCGAGGATCTAACAG
AACTCGCCGTGAAGACTGGCGAACAGTTCATACAGAGTCTTTTACGACTCAATGACAAGAAGAAAATCTTCGTCAACA
TGGTGGAGCACGACACTCTGGTCTACTCCAAAAATGTCAAAGATACAGTCTCAGAAGACCAAAGGGCTATTGAGACTT

dBS F4.1 Primer binding site

TTCAACAAAGGATAATTTCGGGAAACCTCCTCGGATTCCATTGCCC**AGCTATCTGTCACTTCATCGAAAGGACAGTAG**
AAAAGGAAGGTGGCTCCTACAAATGCCATCATTGCGATAAAGGAAAGGCTATCATTCAAGATCTCTGCCGACAGTG
GTCCCAAAGATGGACCCCCACCCACGAGGAGCATCGTGGAAAAAGAAGACGTTCCAACCACGTCTTCAAAGCAAGTGG
ATTGATGTGACATCTCCACTGACGTAAGGGATGACGCACAATCCCACTATCCTTCGCAAGACCCTTCCTCTATATAAG
GAAGTTCATTTCATTTGGAGAGGACACGCTCGAGGAATTCAATGGTGAGCAAGGGCGAGGAGCTGTTCACCGGGGTGG
TGCCCATCCTGGTCGAGCTGGACGGCGACGTAAACGGCCACAAGTTCAGC**GTGTCCGGCGAGGGCGAGGGCGAT**GCCA

dBS R7.1 Primer binding site

CCTACGGCAAGCTGACCCTGAAGTTCATCTGCACCACCGGCAAGCTGCCCGTGCCCTGGCCCACCCTCGTGACCACCT
TCACCTACGGCGTGCAGTGCTTCAGCCGCTACCCCGACCACATGAAGCAGCACGACTTCTTCAAGTCCGCCATGCCCG
AAGGCTACGTCCAGGAGCGCACCATCTTCTTCAAGGACGACGGCAACTACAAGACCCGCGCCGAGGTGAAGTTCGAGG
GCGACACCCTGGTGAACCGCATCGAGCTGAAGGGCATCGACTTCAAGGAGGACGGCAACATCCTGGGGCACAAGCTGG
AGTACAACTACAACAGCCACAACGTCTATATCATGGCCGACAAGCAGAAGAACGGCATCAAGGTGAACTTCAAGATCC
GCCACAACATCGAGGACGGCAGCGTGCAGCTCGCCGACCACTACCAGCAGAACACCCCCATCGGCGACGGCCCCGTGC
TGCTGCCCGACAACCACTACCTGAGCACCCAGTCCGCCCTGAGCAAAGACCCCAACGAGAAGCGCGATCACATGGTCC
TGCTGGAGTTCGTGACCGCCGCCGGGATCACTCTCGGCATGGACGAGCTGTACAAGTAAAGCCCGGATCTCTAGTCTT
CTAGAGTCCTGCTTTAATGAGATATGCGAGACGCCTATGATCGCATGATATTTGCTTTCAATTCTGTTGTGCACGTTG
TAAAAAACCTGAGCATGTGTAGCTCAGATCCTTACCGCCGGTTTCGGTTCATTCTAATGAATATATCACCCGTTACTA
TCGTATTTTTATGAATAATATTCTCCGTTCAATTTACTGATTGTACCCTACTACTTATATGTACAATATTAAAATGAA
AACAATATATTGTGCTGAATAGGTTTATAGCGACATCTATGATAGAGCGCCACAATAACAAACAATTGCGTTTTATTA
TTACAAATCCAATTTTAAAAAAAGCGGCAGAACCGGTCAAACCTAAAAGACTGATTACATAAATCTTATTCAAATTTC
AAAAGGCCCCAGGGGCTAGTATCTACGACACACCGAGCGGCGAACTAATAACGTTCACTGAAGGGAACTCCGGTTCCC
CGCCGGCGCGCATGGGTGAGATTCCTTGAAGTTGAGTATTGGCCGTCCGCTCTACCGAAAGTTACGGCACCATTCAA
CCCGGTCCAGCACGGCGGCCGGGTAACCGACTTGCTGCCCCGAGAATTATGCAGCATTTTTTTGGTGTATGTGGGCCC
CAAATGAAGTGCAGGTCAAACCTTGACAGTGACGACAAATCGTTGGGCGGGTCCAGGGCGAATTTTGCGACAACATGT
CGAGGCTCAGCAGGACCTGCAGCGAATTC

Shaded regions represent: HindIII, 35S Promoter, eGFP, OCS Terminator, EcoRI

Bolded sequences are the degenerate primer binding regions

Bolded cytosines were analysed for methylation, **C** (CG context), **C** (CHG context), **C** (CHH context)

Supplementary Figure S1. DNA sequence containing the 35S promoter, eGFP coding sequence and OCS transcriptional terminator introduced into the pORE 03 backbone (Coutu et al., 2007) via the HindIII and EcoRI restriction sites.

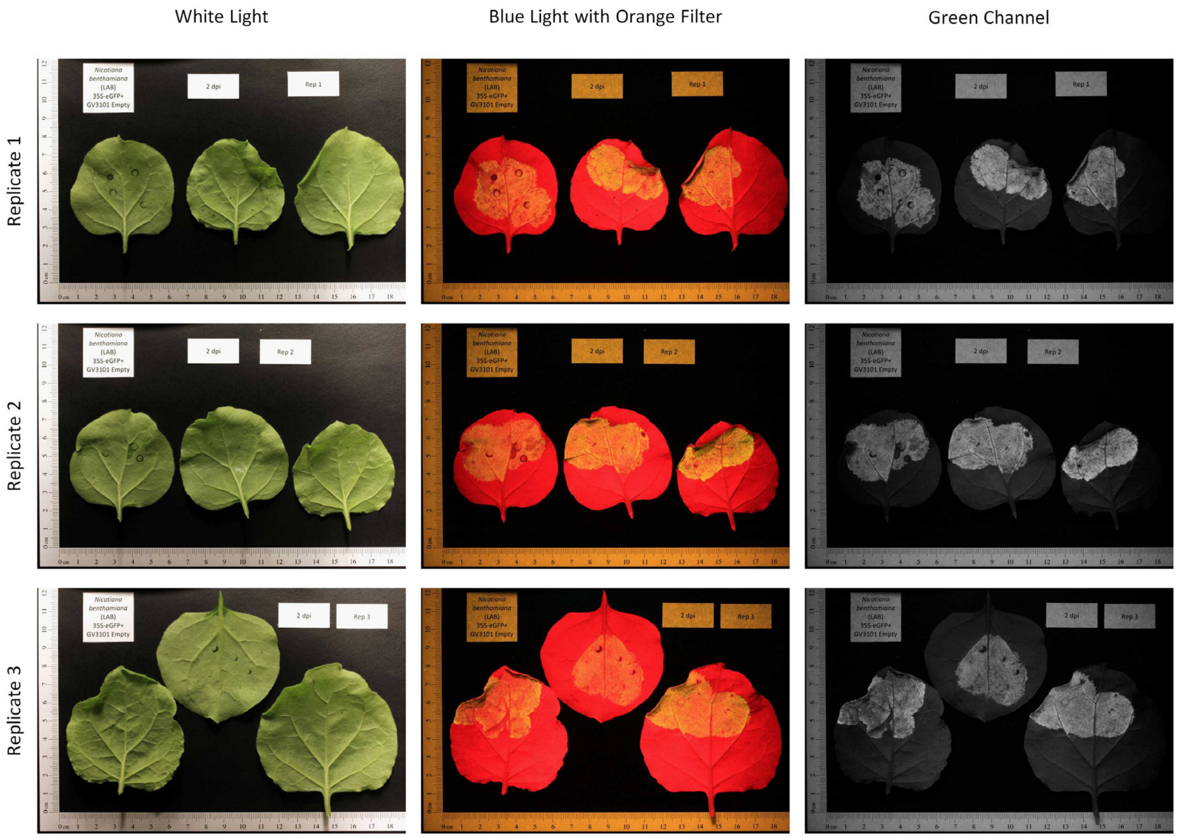

35S-eGFP+GV3101 2 dpi

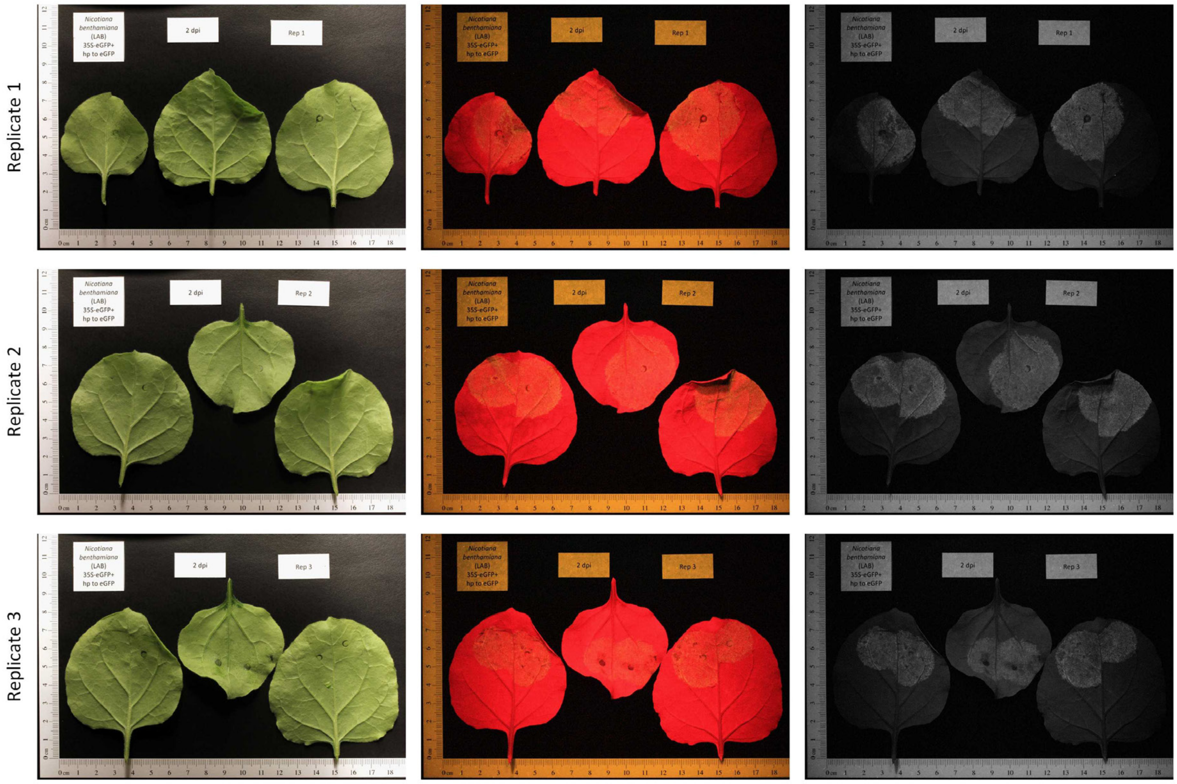

35S-eGFP+hp 2 dpi

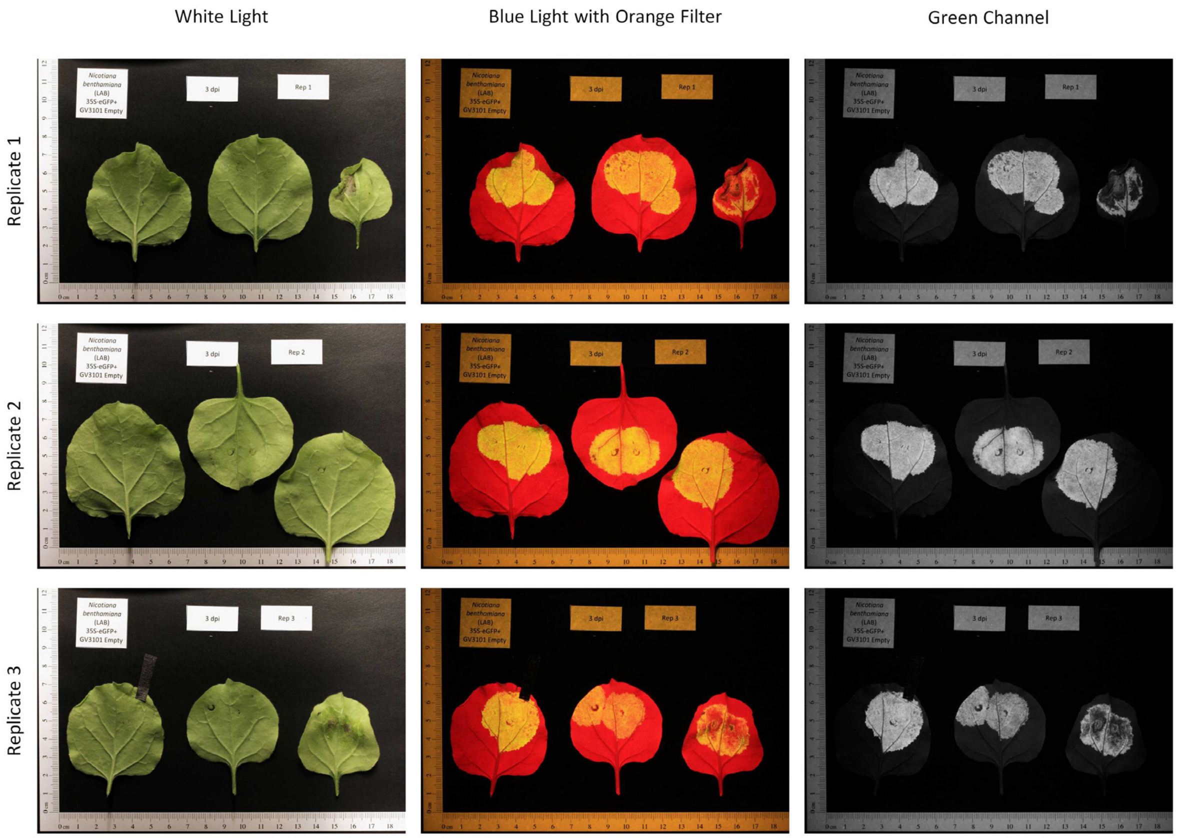

35S-eGFP+GV3101 3 dpi

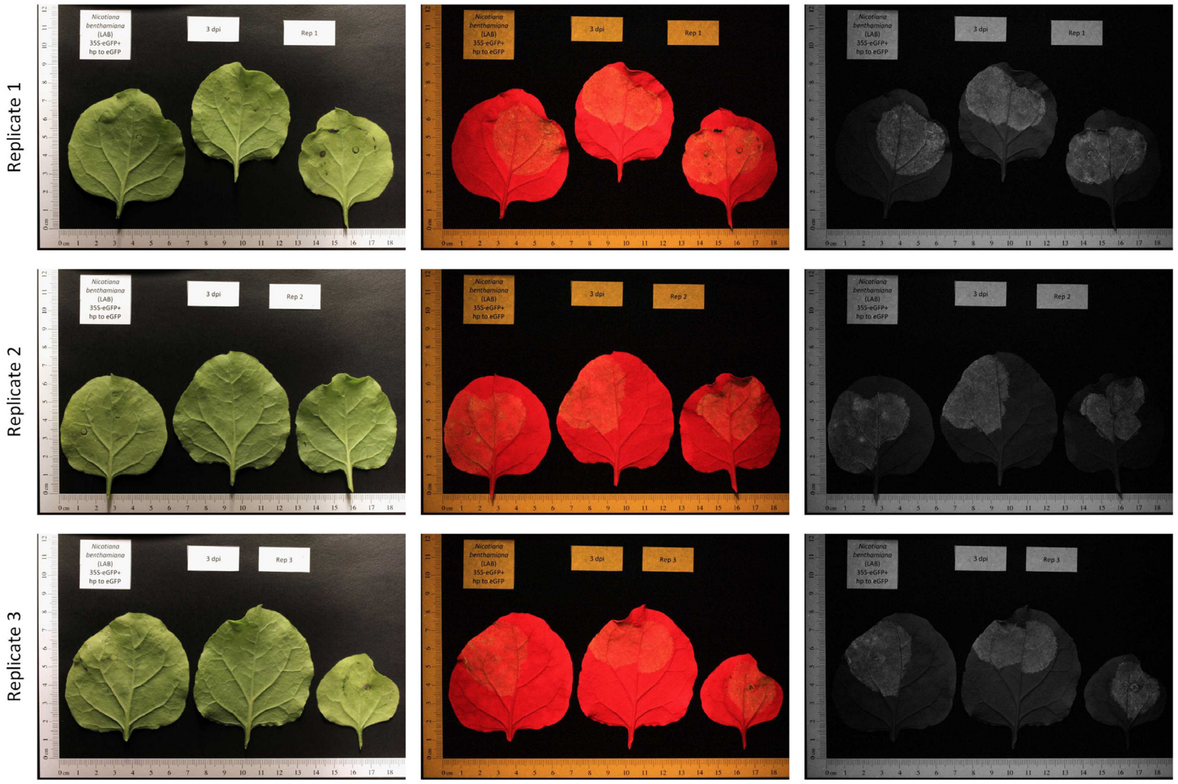

35S-eGFP+hp 3 dpi

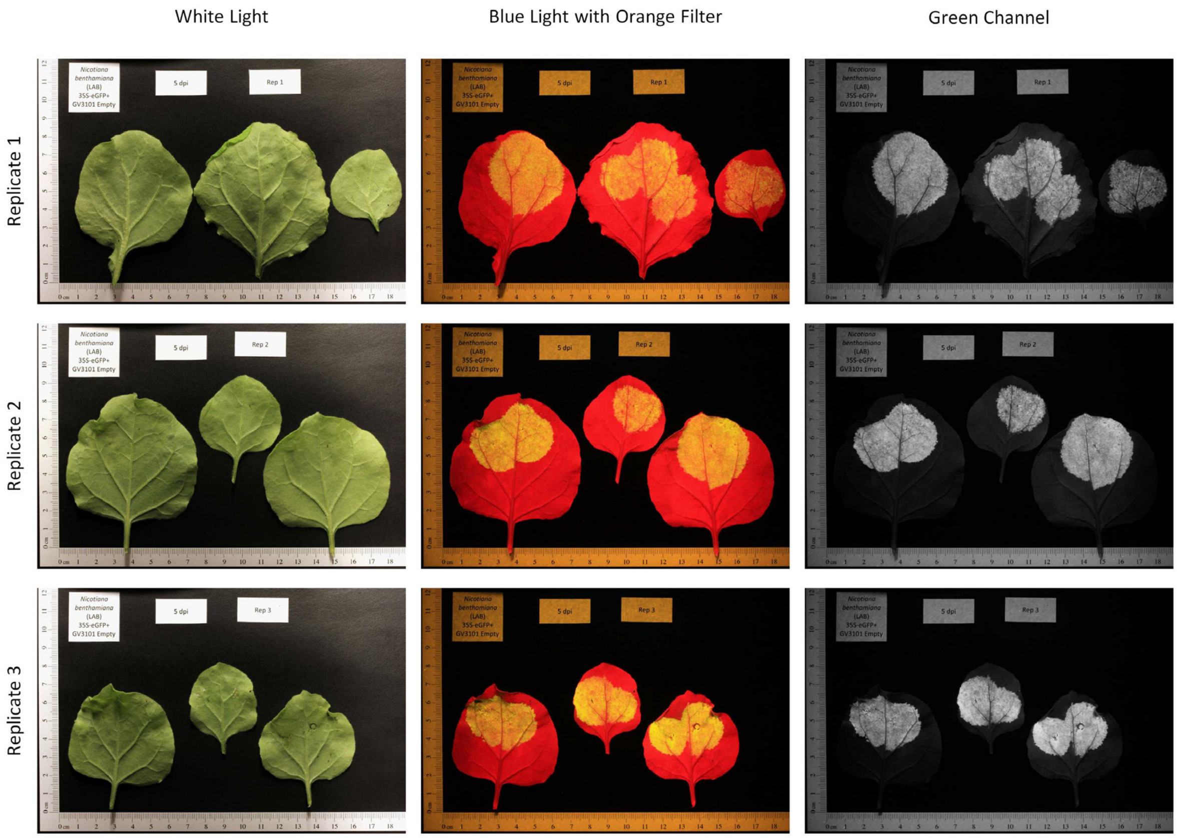

35S-eGFP+GV3101 5 dpi

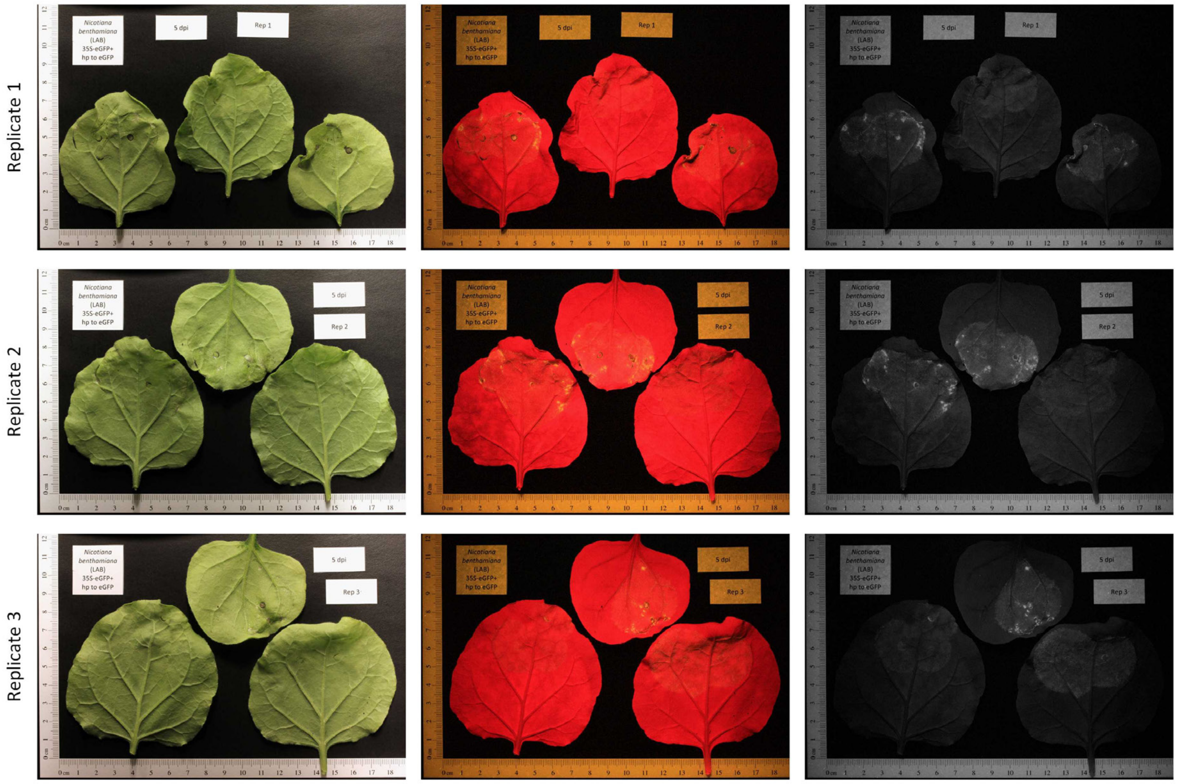

35S-eGFP+hp 5 dpi

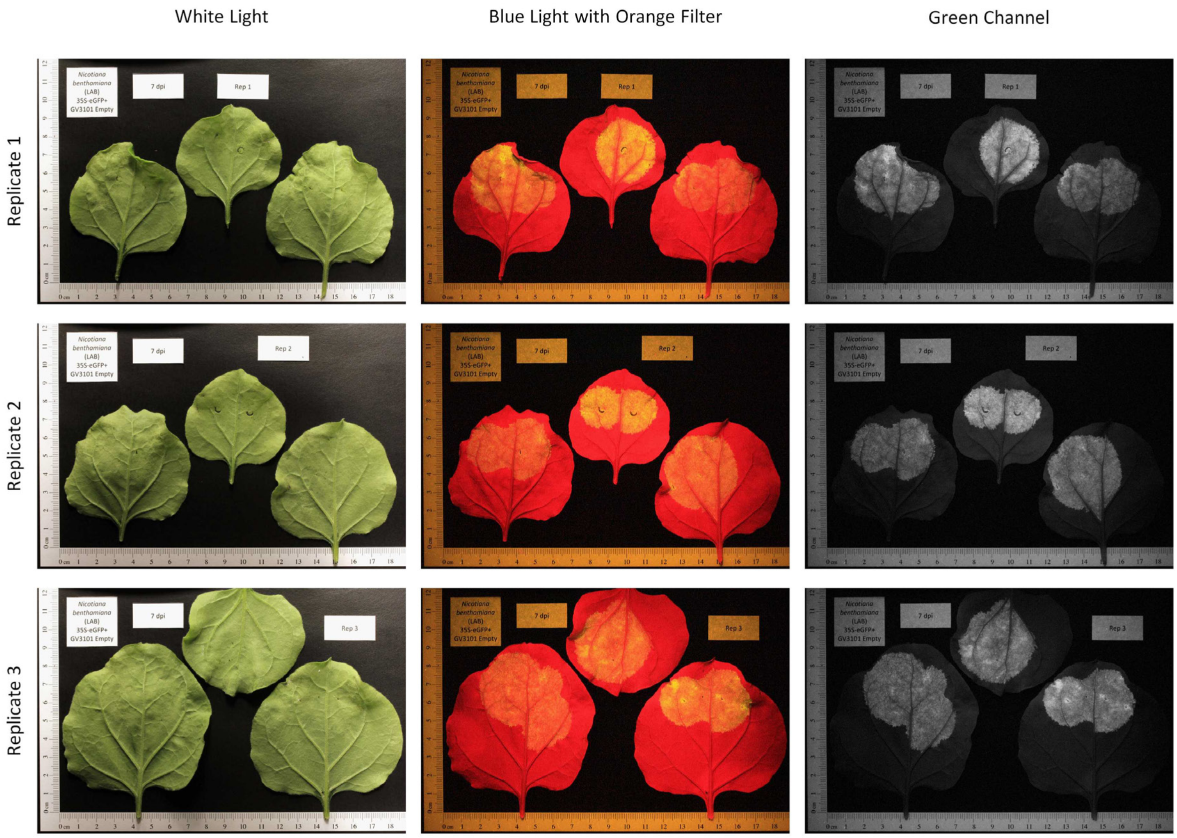

35S-eGFP+GV3101 7 dpi

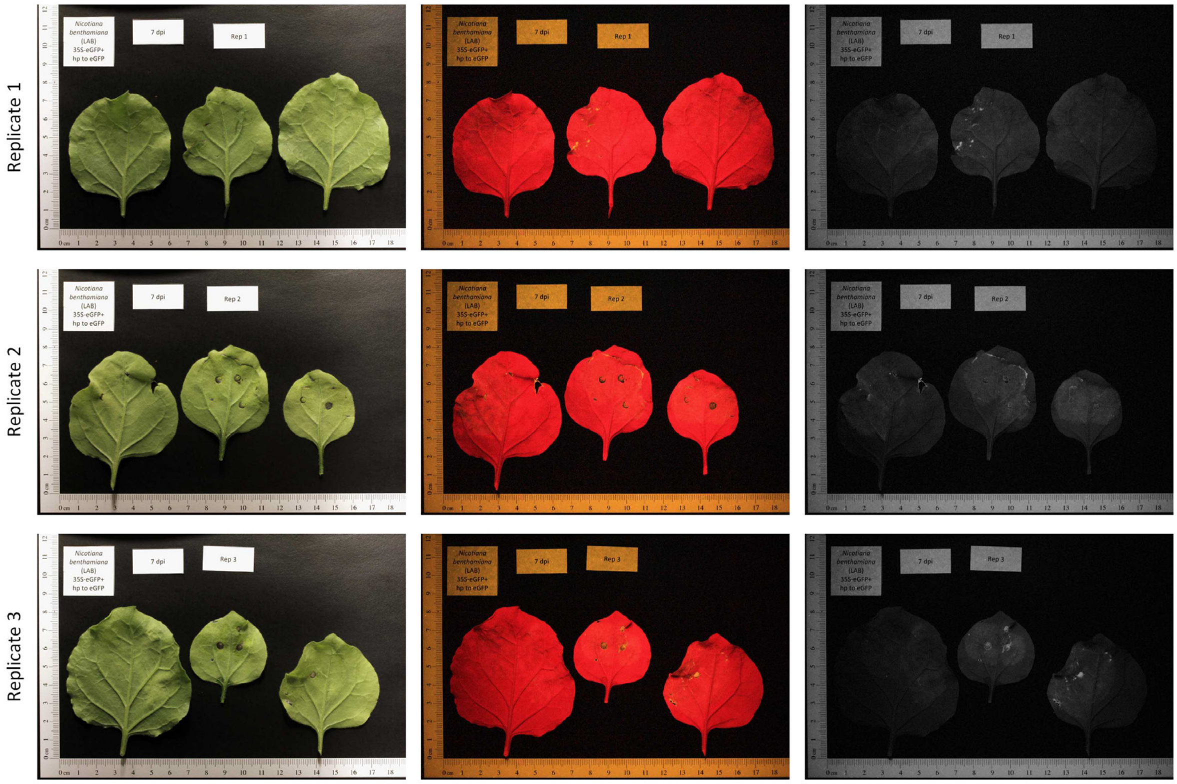

35S-eGFP+hp 7 dpi

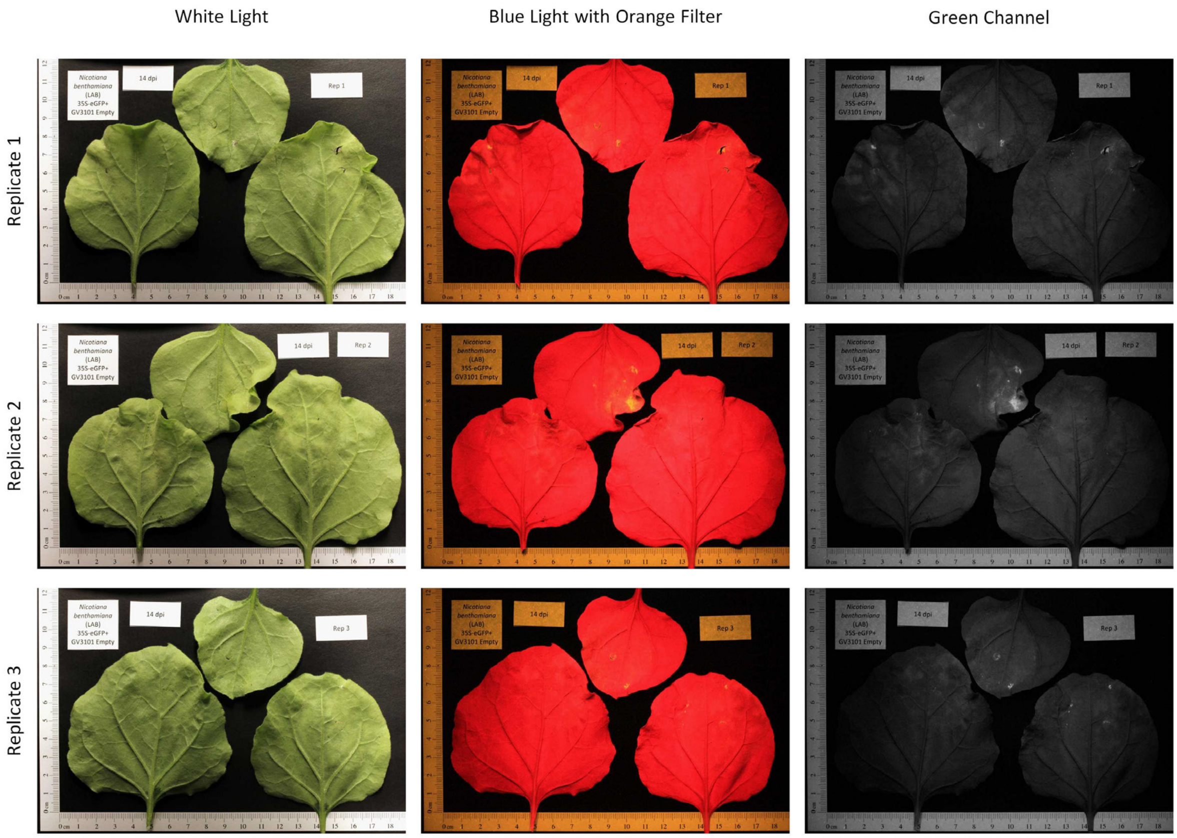

35S-eGFP+GV3101 14 dpi

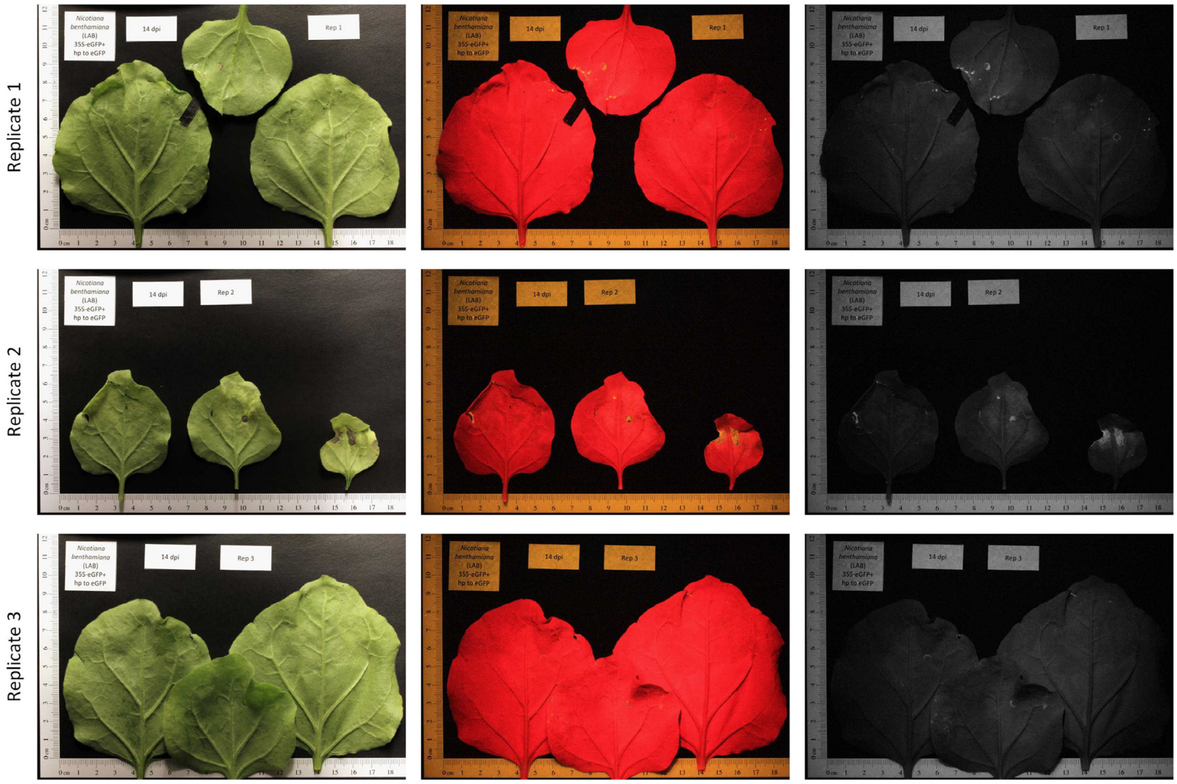

35S-eGFP+hp 14 dpi

White Light | Blue Light with Orange Filter | Green Channel

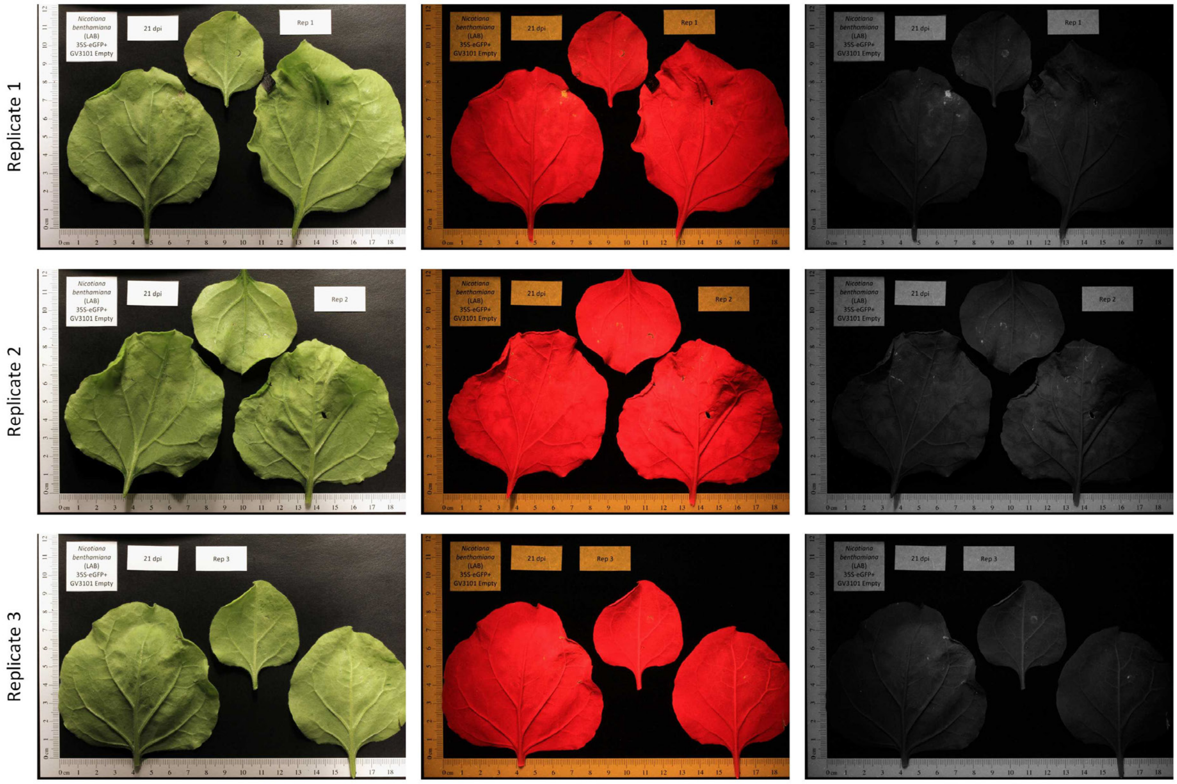

35S-eGFP+GV3101 21 dpi

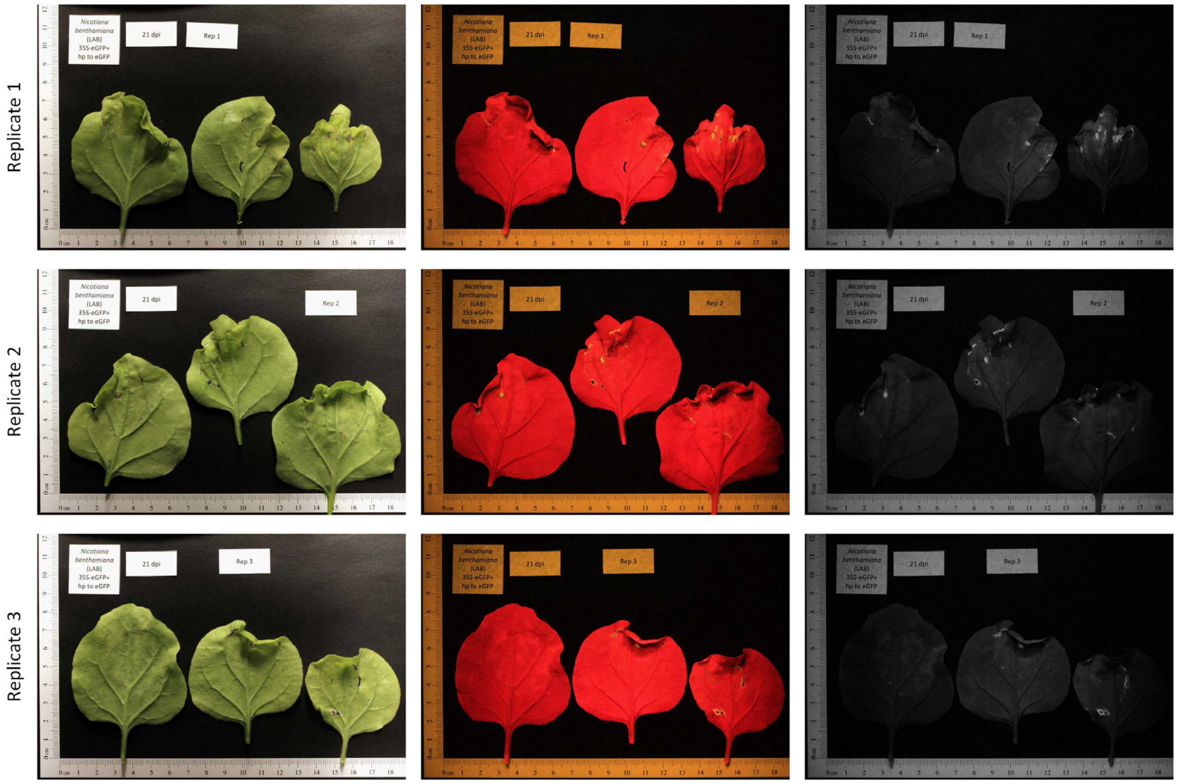

35S-eGFP+hp 21 dpi

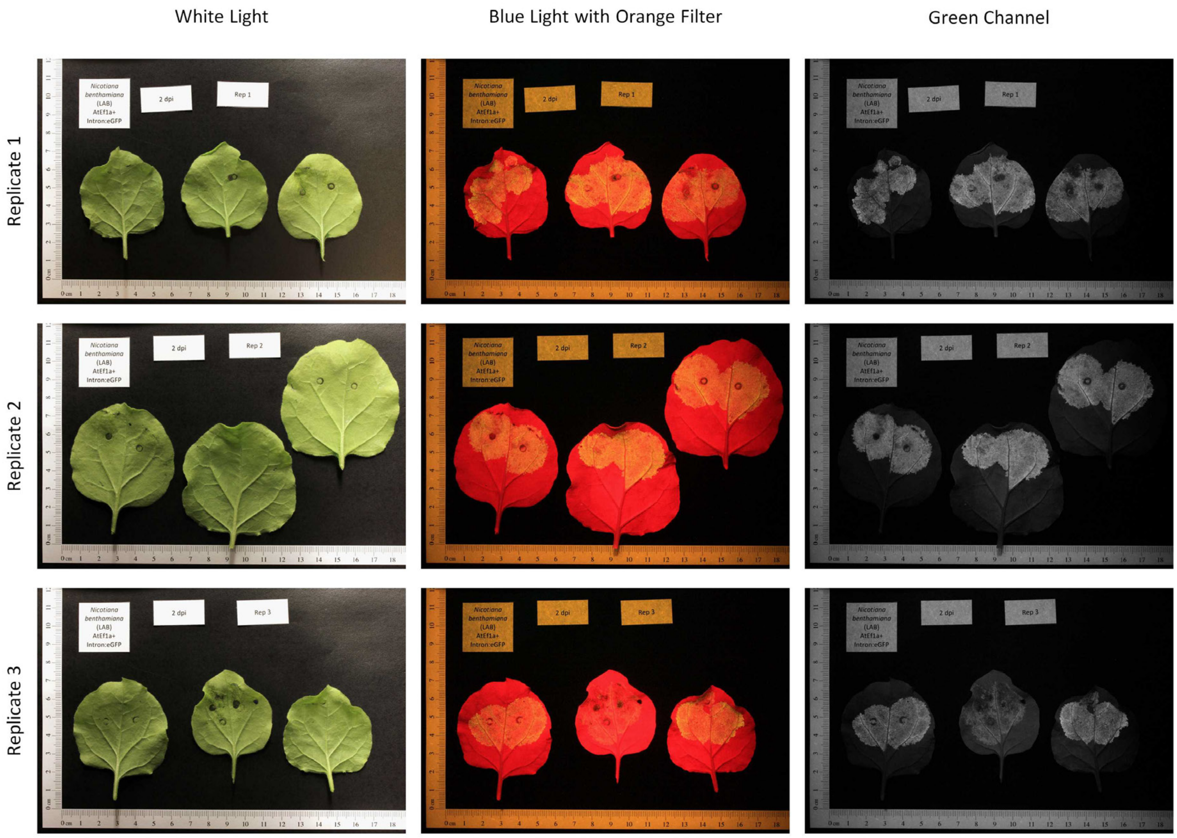

AtEf1α-4 +Intron 2 dpi

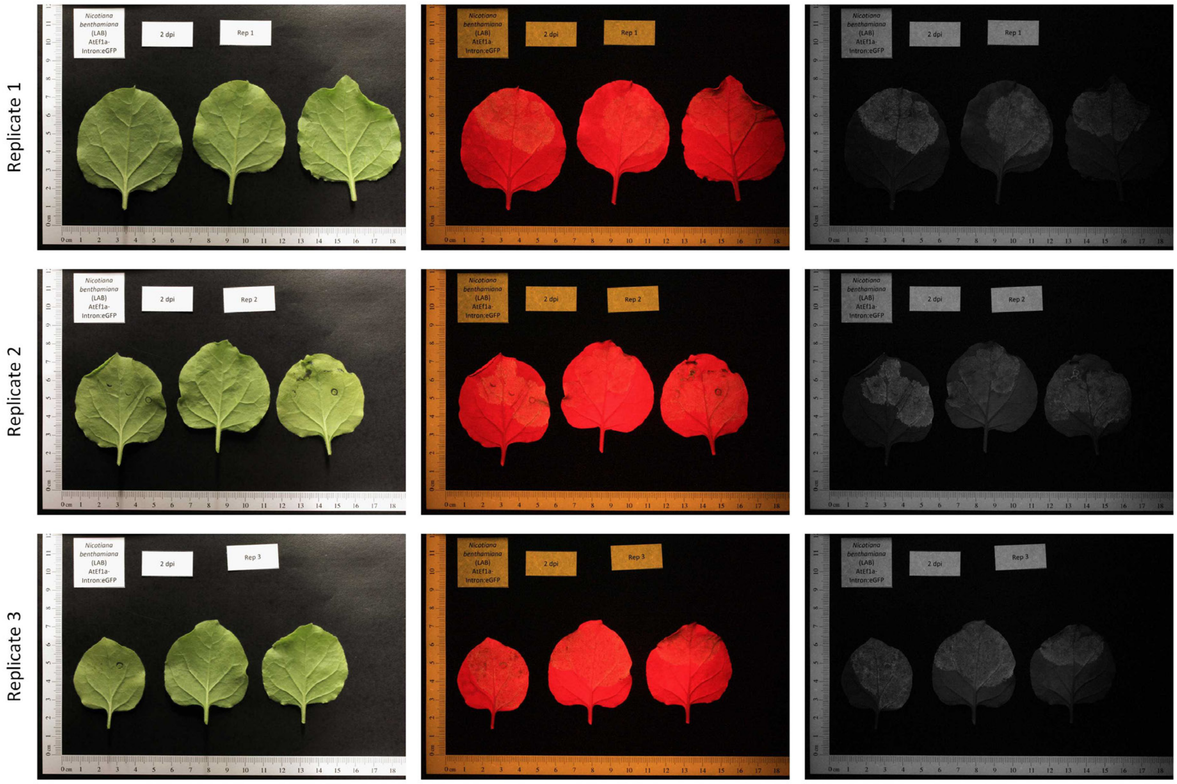

AtEf1α-4 -Intron 2 dpi

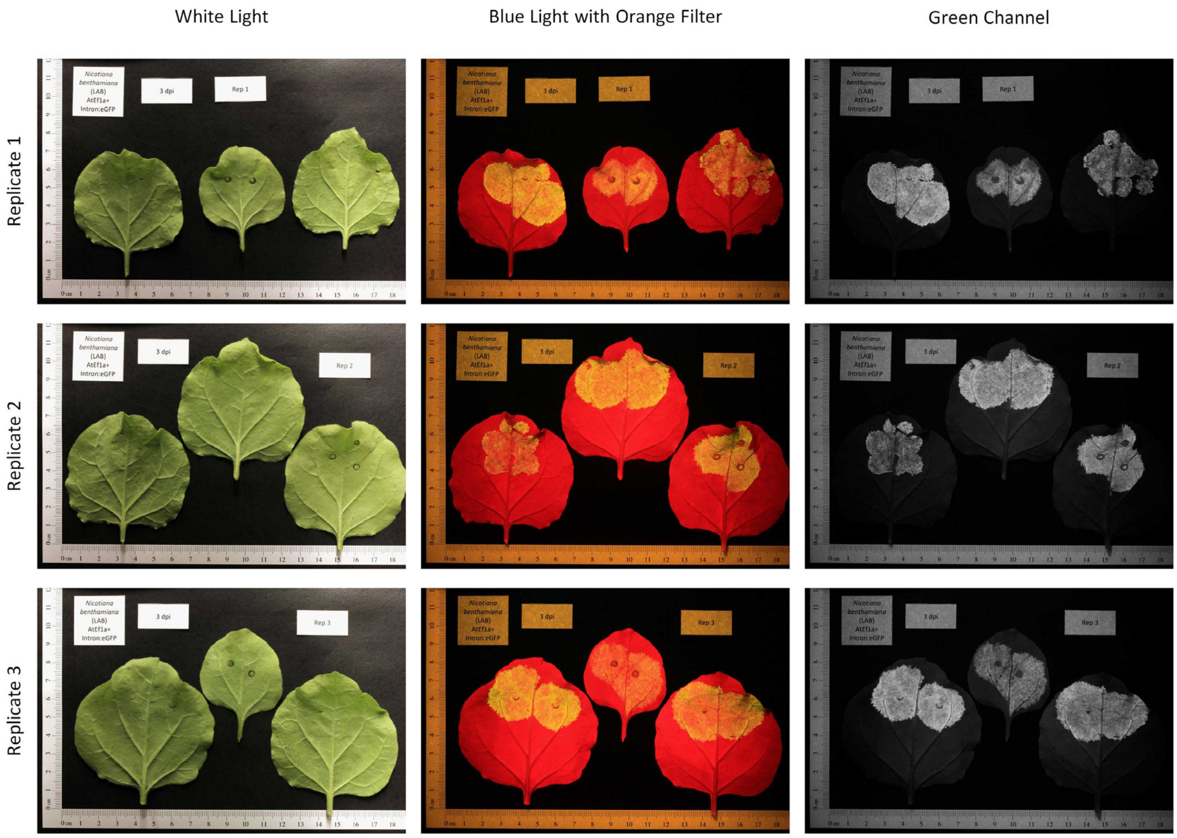

AtEf1α-4 +Intron 3 dpi

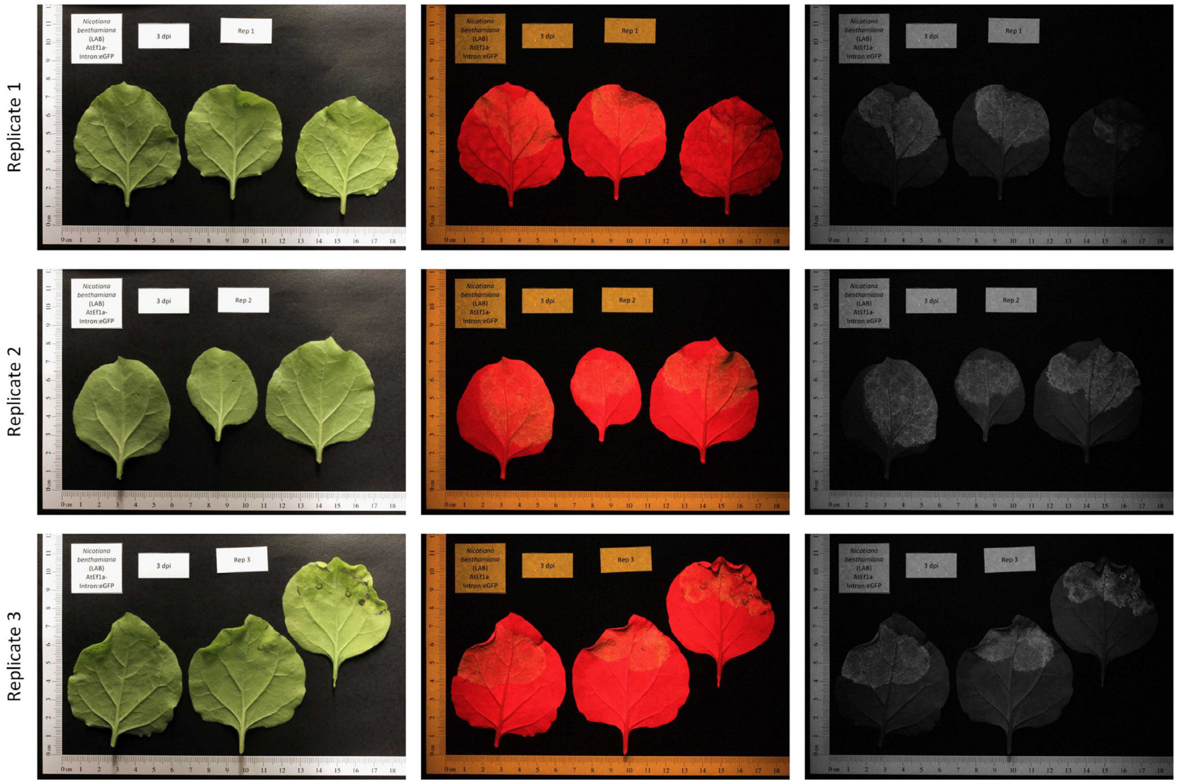

AtEf1α-4 -Intron 3 dpi

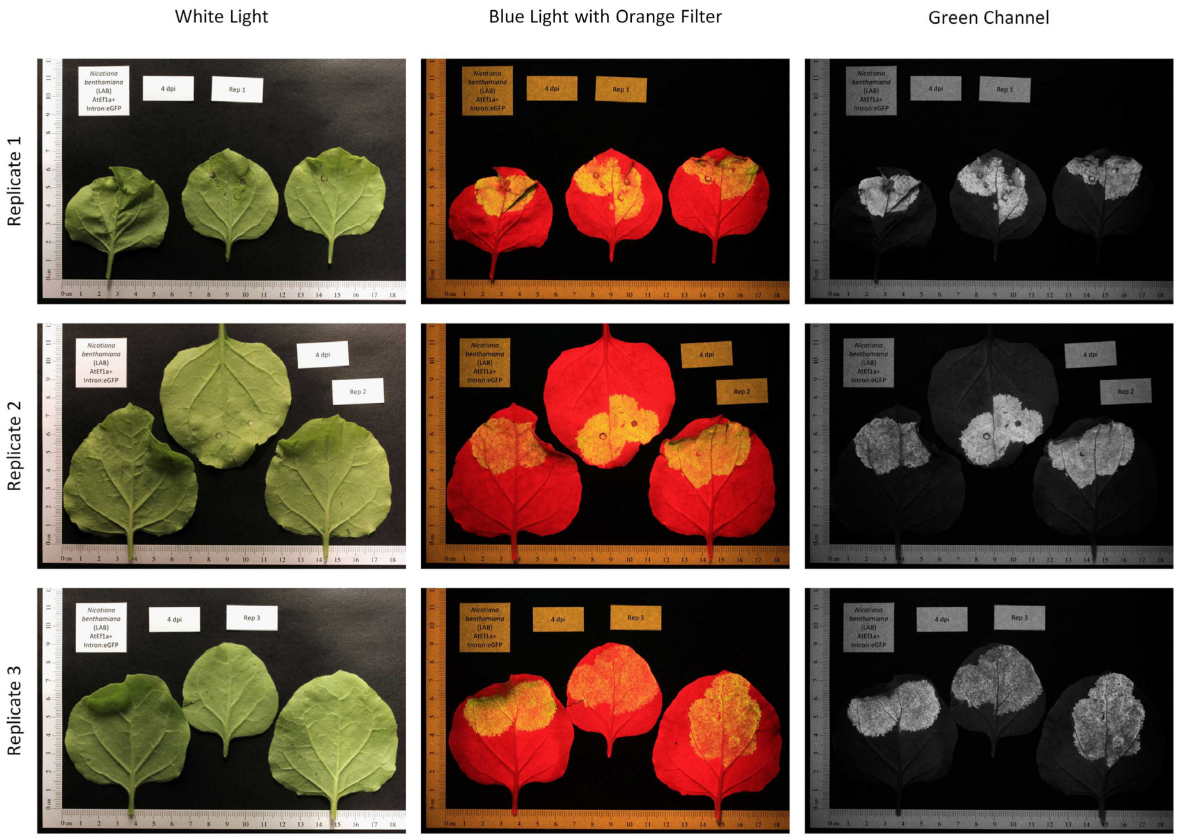

AtEf1α-4 +Intron 4 dpi

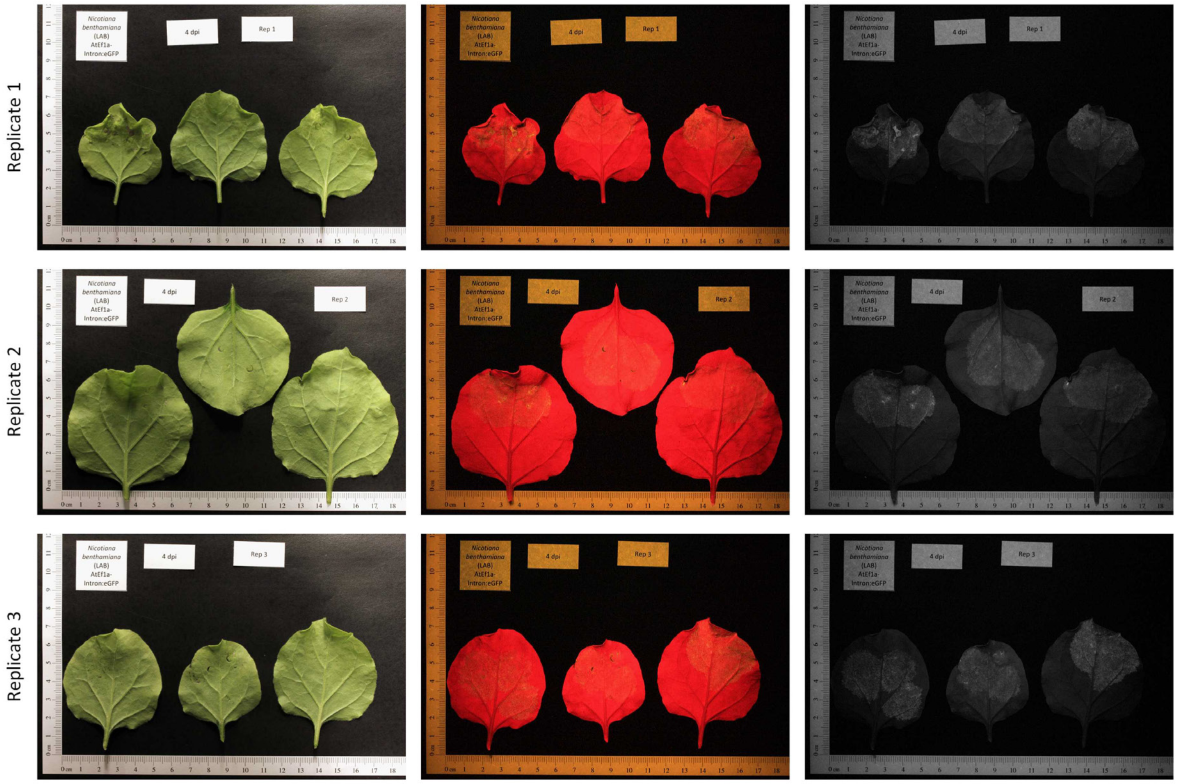

AtEf1α-4 -Intron 4 dpi

White Light | Blue Light with Orange Filter | Green Channel

Replicate 1
Replicate 2
Replicate 3

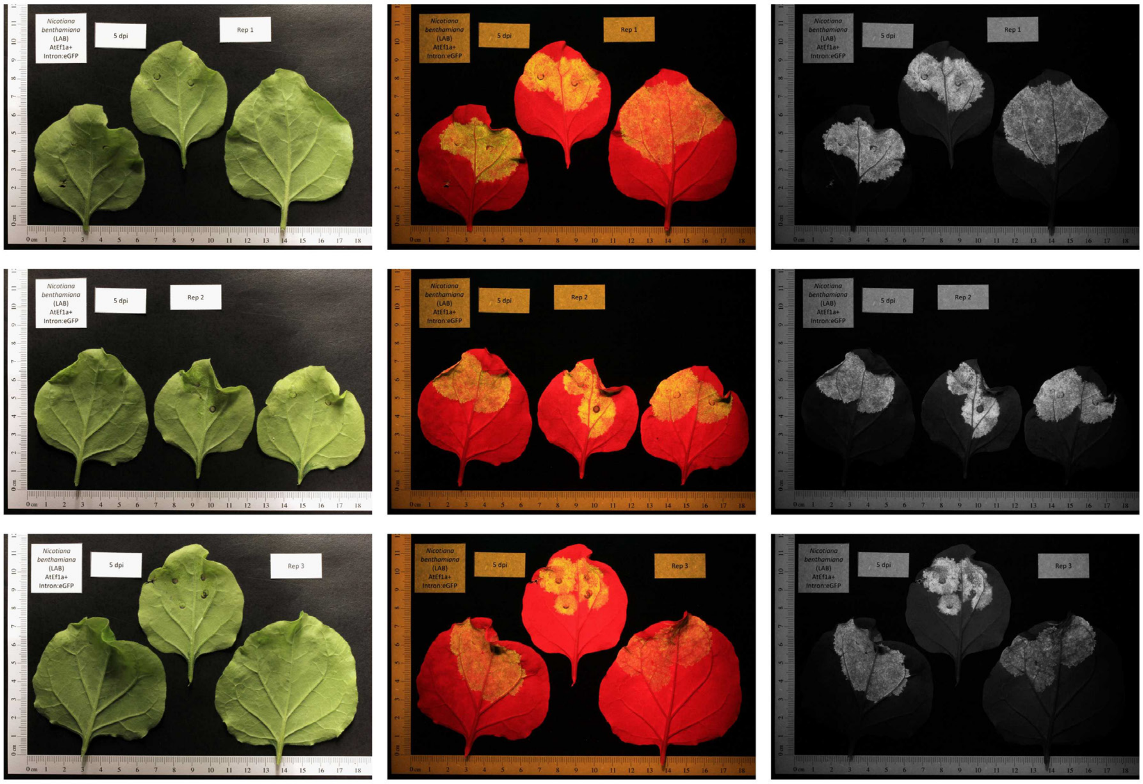

AtEf1α-4 +Intron 5 dpi

Replicate 1
Replicate 2
Replicate 3

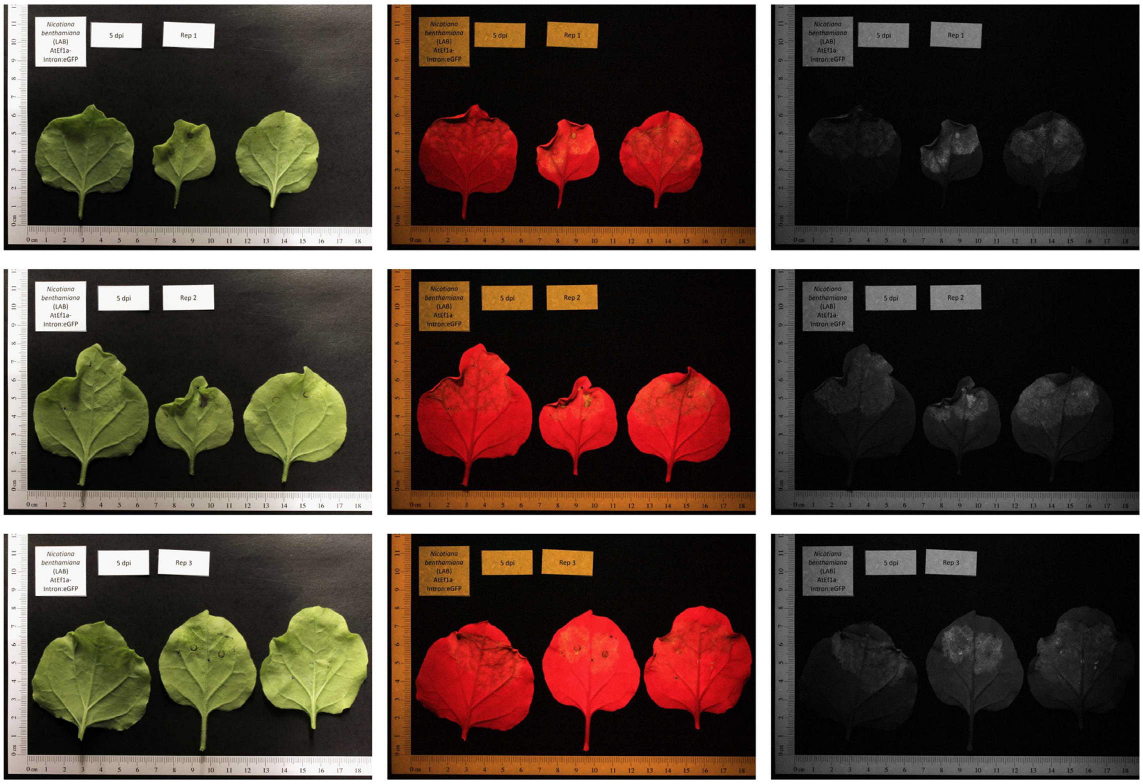

AtEf1α-4 -Intron 5 dpi

White Light Blue Light with Orange Filter Green Channel

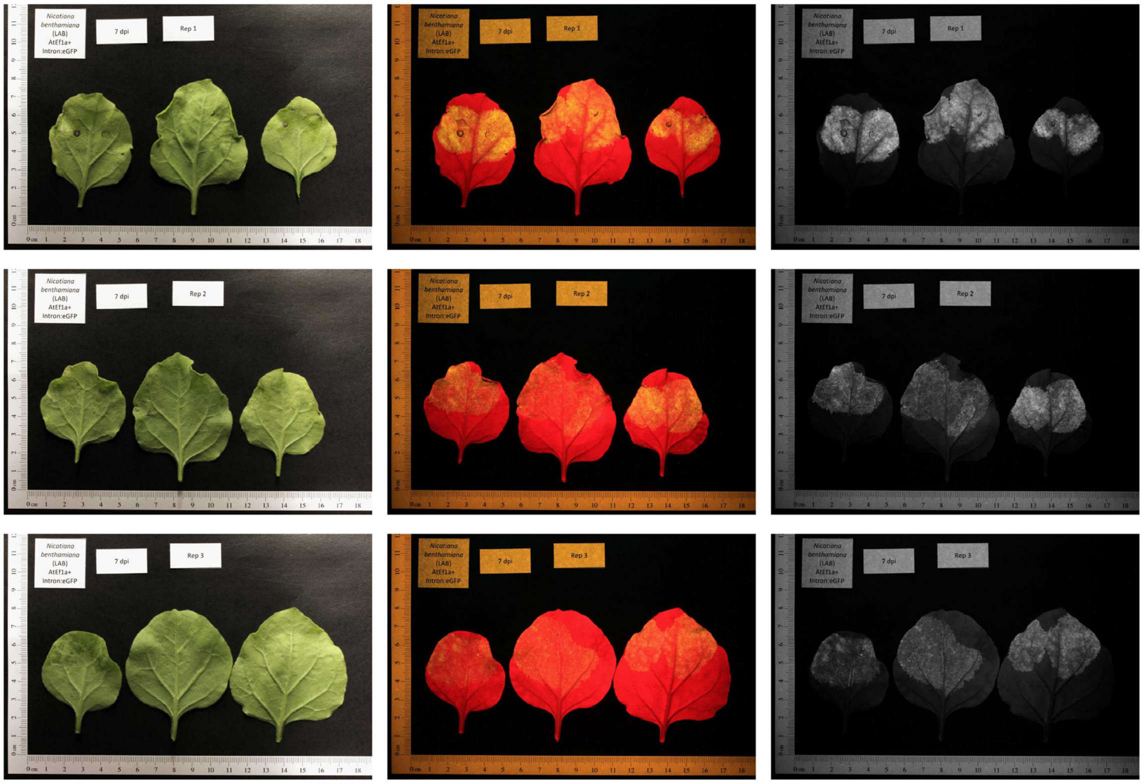

AtEf1α-4 +Intron 7 dpi

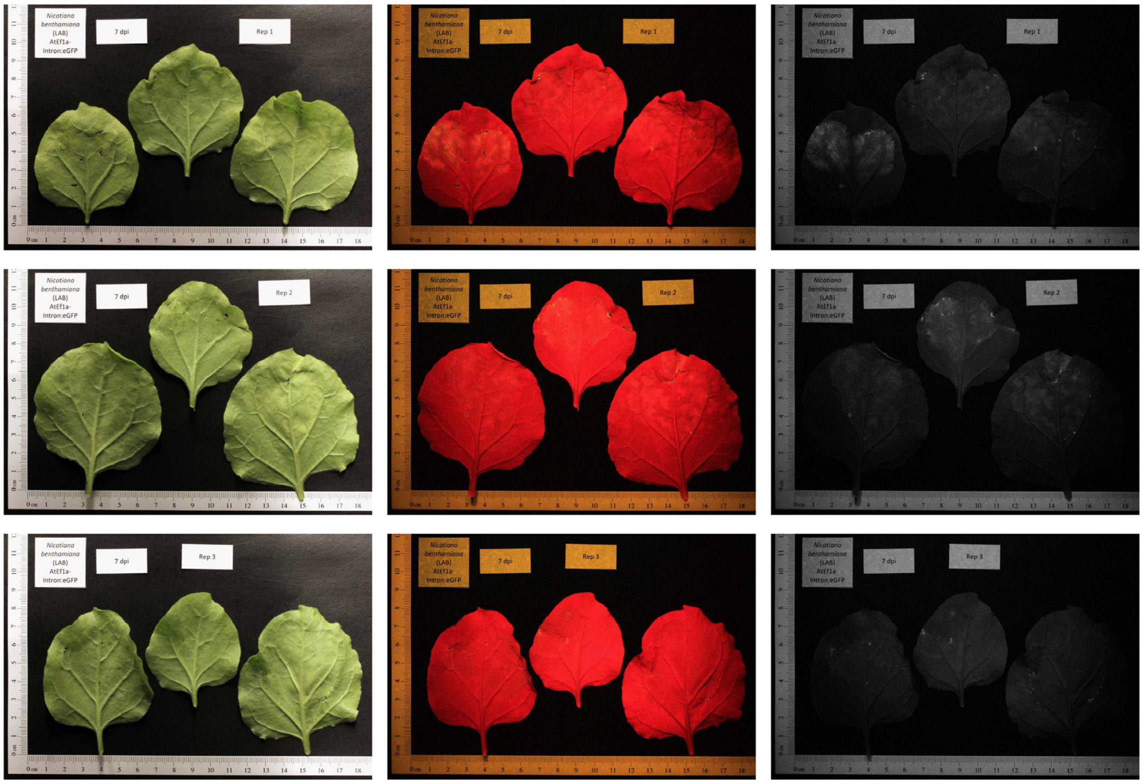

AtEf1α-4 -Intron 7 dpi

Supplementary Figure S2. (Pages 7-12) Images of *N. benthamiana* leaves infiltrated with 35S-eGFP (top panel) and infiltrated with 35S-eGFP+hp (bottom panel). **(Pages 13-17)** Images of *N. benthamiana* leaves infiltrated with AtEF1α-A4+Intron-eGFP (top panel) and infiltrated with AtEF1α-A4–Intron-eGFP (bottom panel). Fluorescent images were captured by applying the Dark Reader Hand Lamp HL32T (Clare Chemical) using the Canon EOS 550D DLSR camera affixed with an EF-S 60 mm lens and a Hoya HMC O(G) amber filter blocking blue light transmission. Images were then converted to black-and-white and subjected to ImageJ analysis as described.

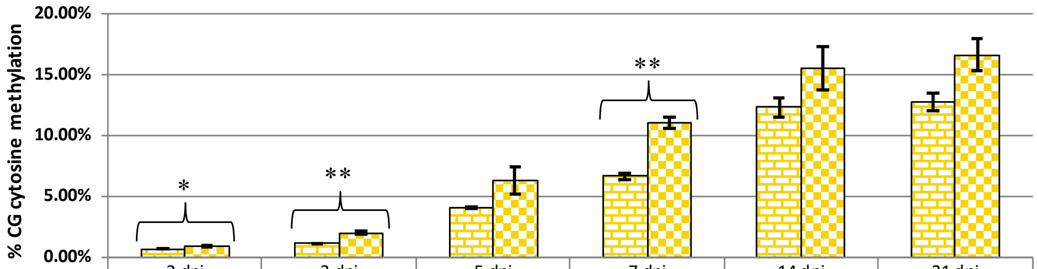

	2 dpi	3 dpi	5 dpi	7 dpi	14 dpi	21 dpi
- hp	0.67%	1.07%	4.06%	6.62%	12.30%	12.71%
+ hp	0.91%	1.96%	6.30%	11.06%	15.51%	16.61%

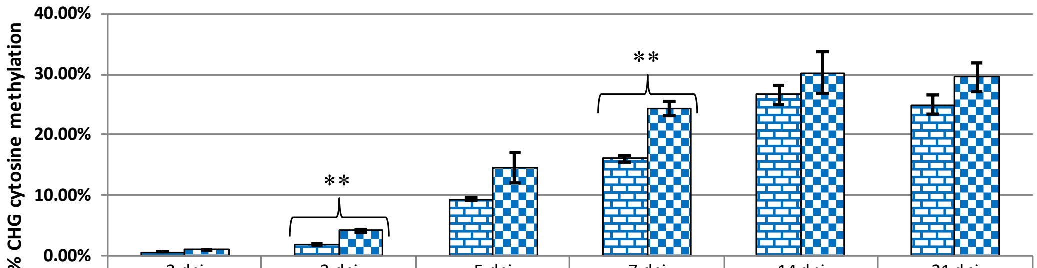

	2 dpi	3 dpi	5 dpi	7 dpi	14 dpi	21 dpi
- hp	0.70%	1.95%	9.40%	16.11%	26.62%	24.99%
+ hp	1.00%	4.19%	14.64%	24.37%	30.23%	29.54%

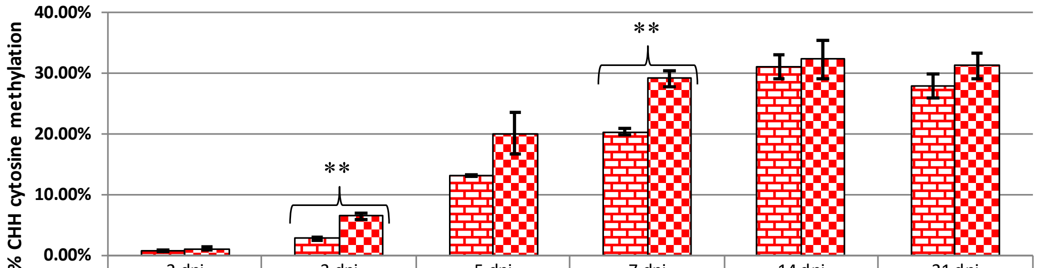

	2 dpi	3 dpi	5 dpi	7 dpi	14 dpi	21 dpi
- hp	0.77%	2.87%	13.10%	20.33%	31.16%	27.94%
+ hp	1.16%	6.47%	20.16%	29.16%	32.36%	31.26%

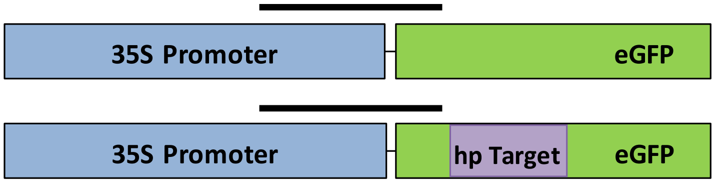

Supplementary Figure S3. Cytosine context specific methylation analysis comparison in the time course of the 35S-eGFP transgene with and without a hairpin. Comparisons carried out by ANOVA. Statistically significant differences, **P* < 0.05, ***P* < 0.01. n = 3 biological replicates, means ± SEM. The black bar in the gene cartoon represents the 418 bp region analysed by bisulfite PCR.

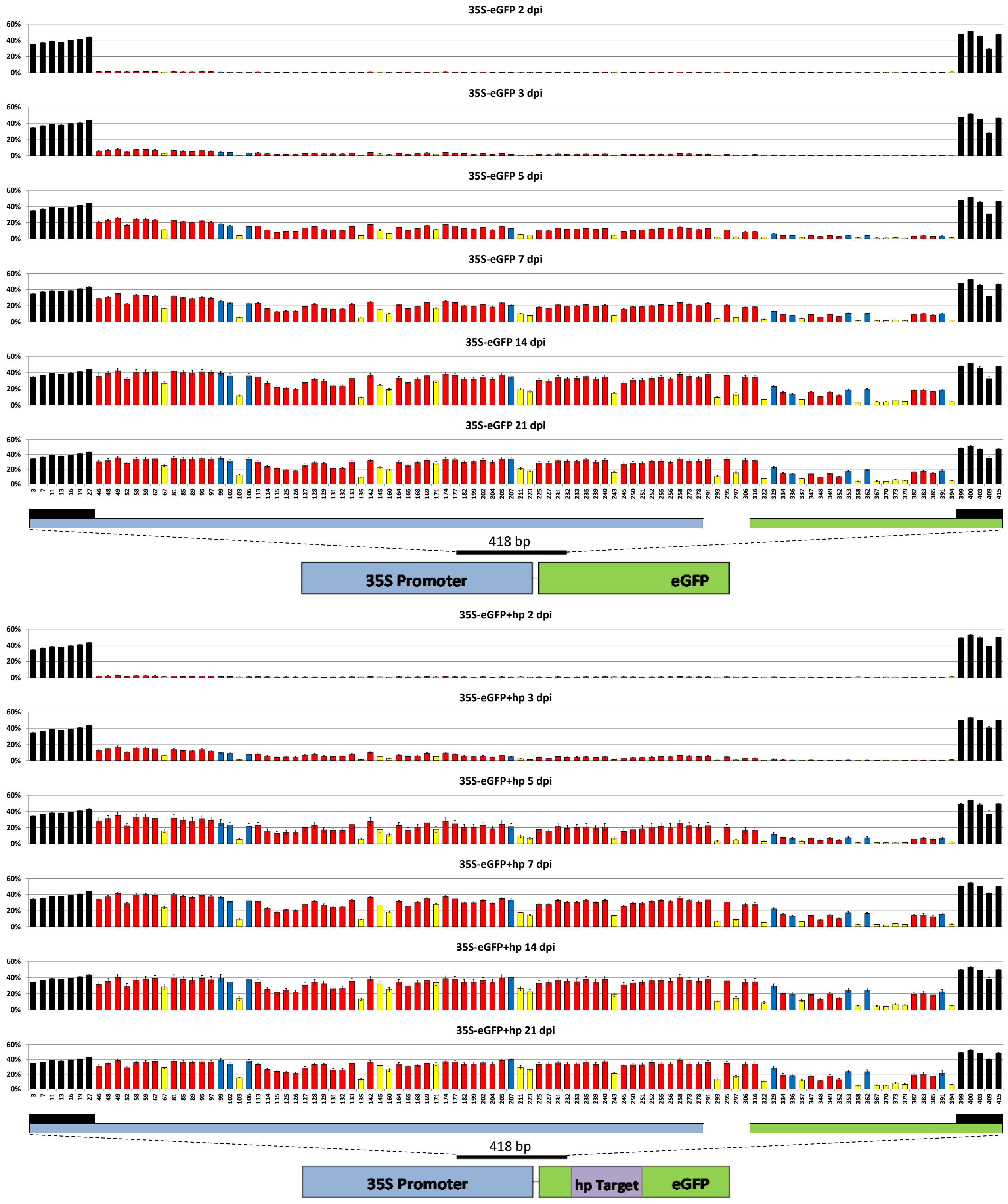

Supplementary Figure S4. Percentage cytosine methylation in the T-DNA of the 35S-eGFP transgene with and without the hpRNA. The black bar represents a 418 bp region analysed by bisulfite PCR. The blue region represents the 35S promoter, green region is the eGFP coding region, purple region is region targeting eGFP by hpRNA and the black region is the degenerate cytosine sites of the primers.

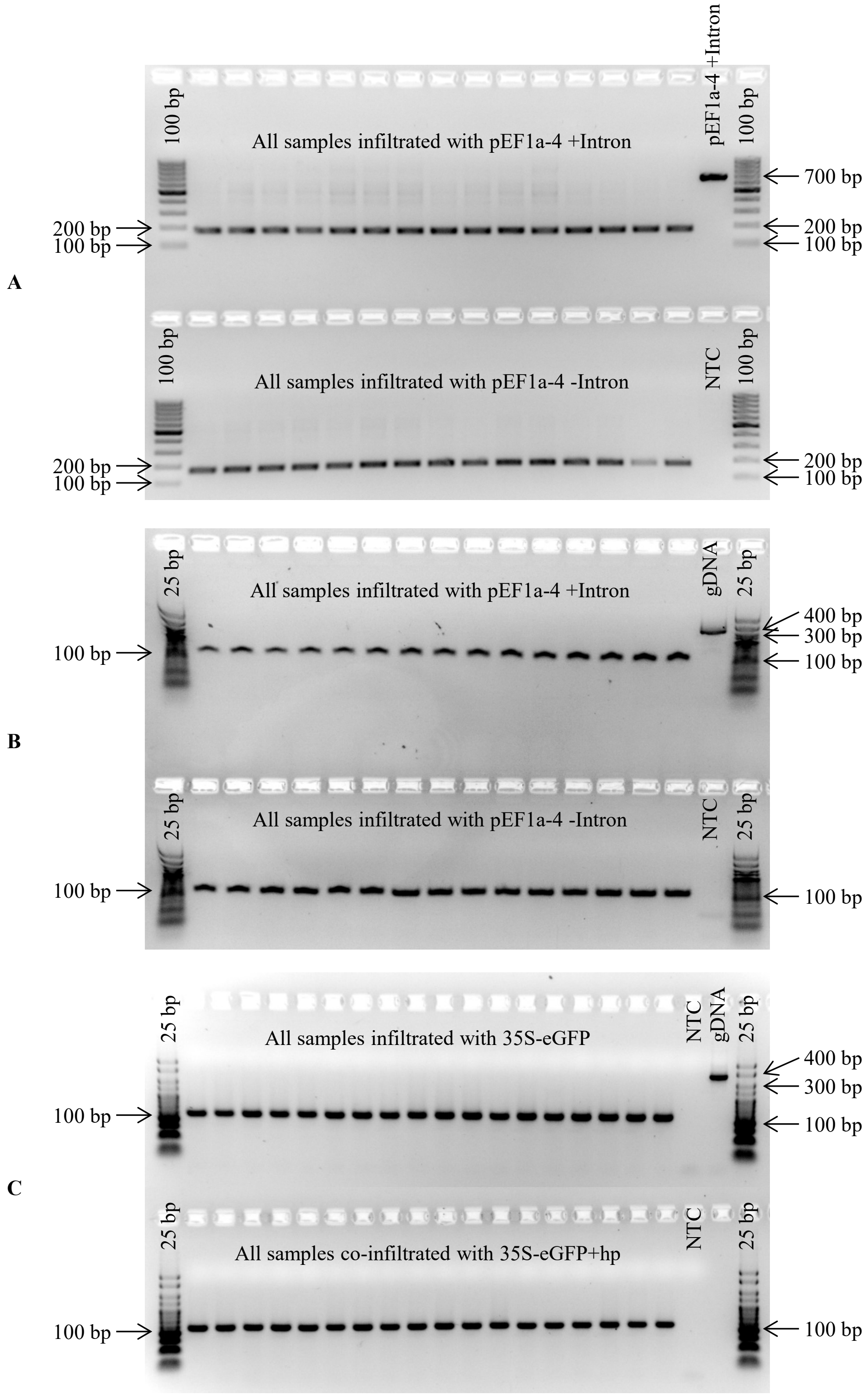

Supplementary Figure S5. (A) RT-PCR using primers EF1a TSS F→dBS R7.1 to test intron processing. Expected sizes: processed 176 bp, unprocessed 675 bp. Diluted minipreparation of the pEF1a-4 +Intron plasmid was used as a positive control for the unprocessed state. 100 bp DNA Ladder (GeneRuler). **(B and C)** RT-PCR using primers NbL23 qPCR F→NbL23 qPCR R to test for residual gDNA contamination after DNaseI treatment of RNA. Expected sizes: cDNA 110 bp, gDNA 338 bp. Hyperladder 25bp (Bioloine).

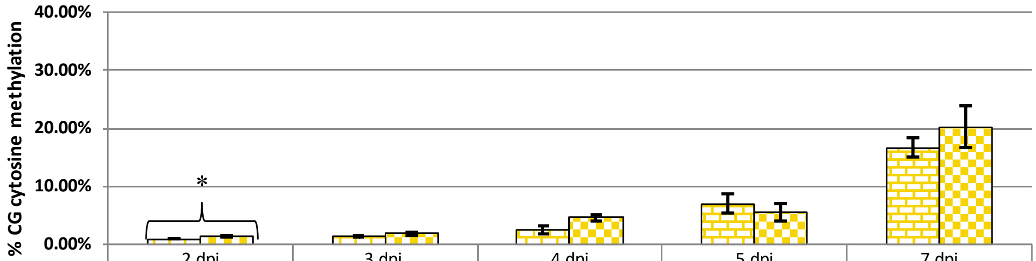

	2 dpi	3 dpi	4 dpi	5 dpi	7 dpi
+ Intron	0.87%	1.35%	2.43%	7.01%	16.63%
- Intron	1.42%	1.82%	4.55%	5.44%	20.27%

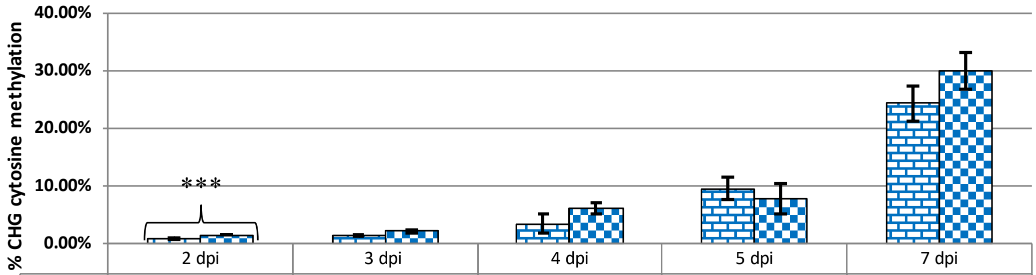

	2 dpi	3 dpi	4 dpi	5 dpi	7 dpi
+ Intron	0.82%	1.35%	3.38%	9.48%	24.23%
- Intron	1.38%	2.08%	6.08%	7.70%	29.83%

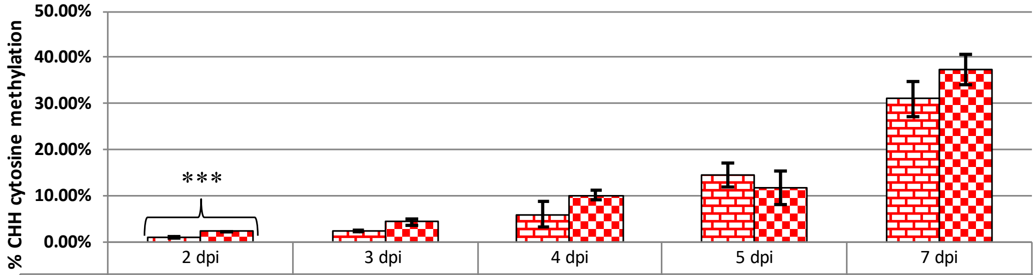

	2 dpi	3 dpi	4 dpi	5 dpi	7 dpi
+ Intron	1.06%	2.36%	6.00%	14.57%	31.00%
- Intron	2.33%	4.34%	10.20%	11.71%	37.34%

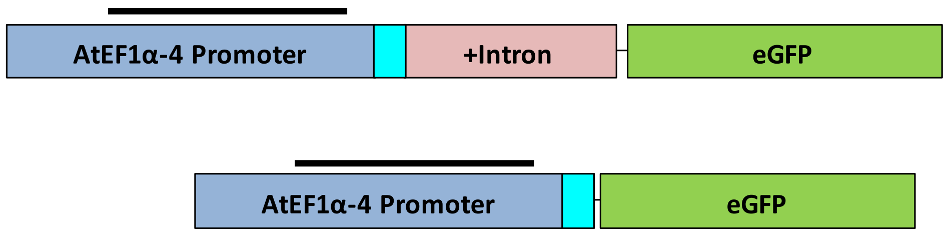

Supplementary Figure S6. Cytosine context specific methylation analysis comparison in the time course of the *AtEF1α-A4* promoter regulating eGFP with and without the 5'UTR intron. Comparisons carried out by ANOVA. Statistically significant differences, **P* < 0.05, ****P* < 0.001. n = 3 biological replicates, means ± SEM. The black bar in the gene cartoon represents the 546 bp region analysed by bisulfite PCR.

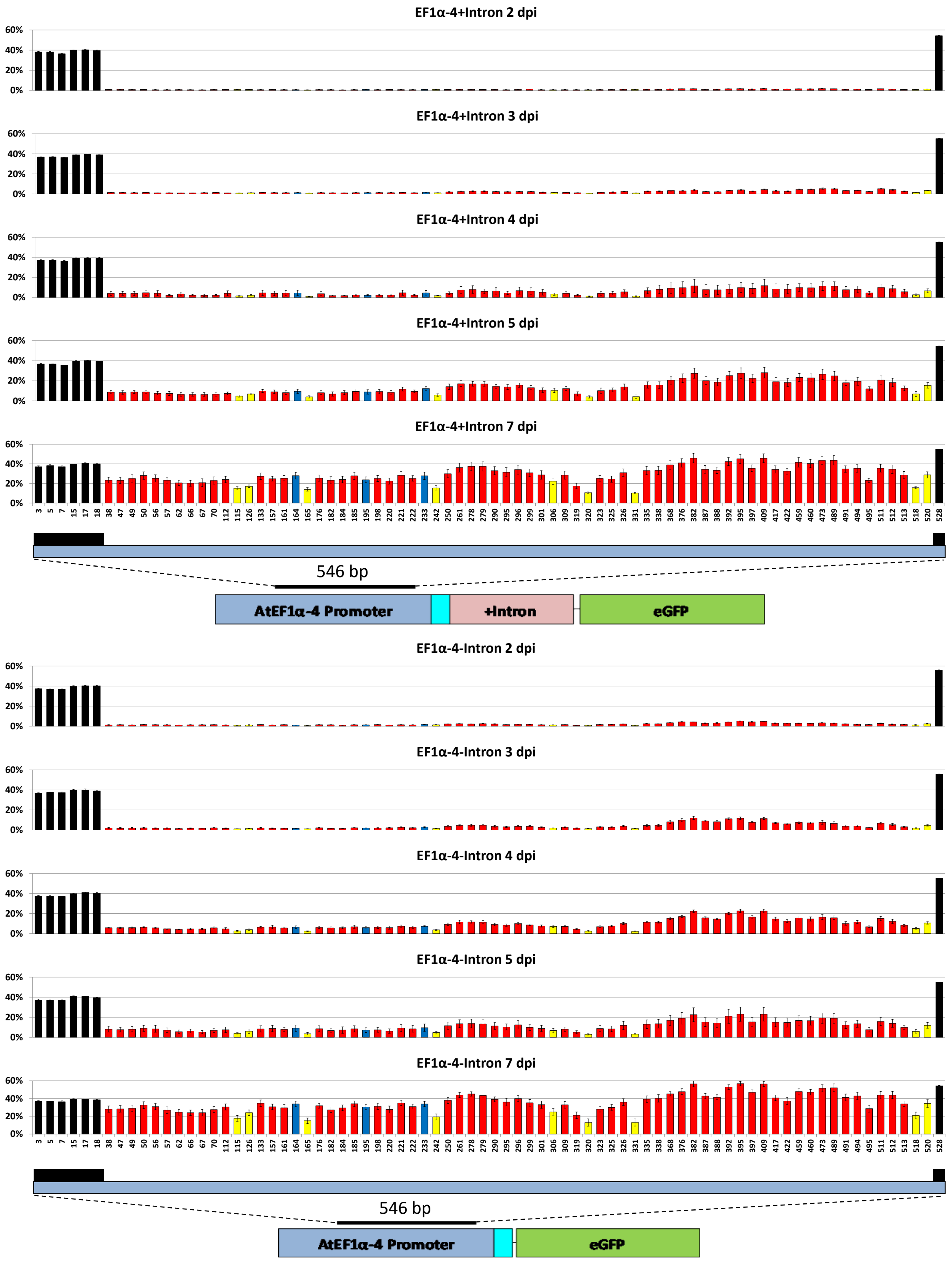

Supplementary Figure S7. Percentage cytosine methylation in the T-DNA of the AtEF1α-eGFP transgene with and without the intron. The black bar represents a 546 bp region analysed by bisulfite PCR. The blue region represents the AtEF1α promoter and the black region is the degenerate cytosine sites of the primers.

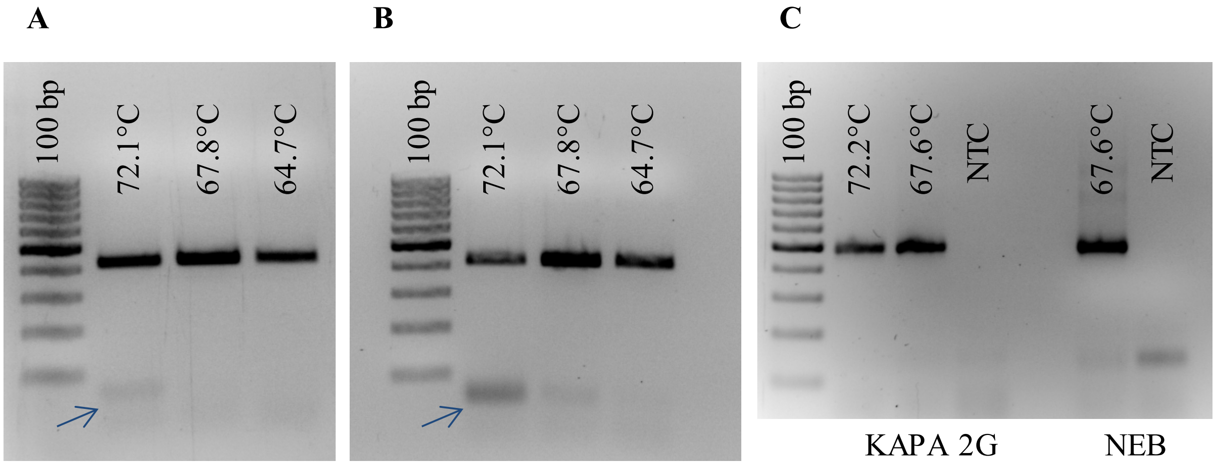

Supplementary Figure S8. Gel electrophoresis showing amplification intensity when differing PCR extension temperatures are used with bisulfite DNA as the template. **(A)** Amplification using dBS F4.1 and dBS R7.1 primers with pooled bisulfite converted DNA (2-21 dpi+hp) as template **(B)** Amplification using dBS F4.1 and dBS R7.1 primers with bisulfite converted DNA from the 2 dpi+hp time point as template. **(C)** Amplification using dBS F4.1 Illumina and dBS R7.1 Illumina primers from the 14 dpi+hp time point as template, also presented in Figure 4 B. Arrows indicate primer-dimers. 100 bp DNA Ladder (GeneRuler).
